# Clinical Course of Kidney Injury Associated With Specific Red Yeast Rice–Containing Supplements

**DOI:** 10.1016/j.ekir.2026.107107

**Published:** 2026-09-30

**Authors:** Yohei Doi, Maki Shinzawa, Isao Matsui, Masaomi Nangaku, Yoshitaka Isaka, Collaborators, Masami Ono, Yuriko Yonekura, Yuki Nishizawa, Yuri Kasagi, Masahiro Azuma, Ryousuke Aoki, Koji Inagaki, Keiji Kono, Akihiro Tojo, Hideyuki Katori, Ken-ichi Miyoshi, Satoru Shichijo, Akiko Torii, Reina Miyazaki, Sinsuke Motegi, Yasutaka Kamikawa, Emi Kitashoji, Takehiko Tokura, Yoshikazu Nemoto, Masahito Baba, Risshi Kudo, Atsushi Saito, Ryuta Fujimura, Takashi Araki, Hiraku Yoshida, Takanori Miyazaki, Daiki Nagawa, Yosuke Osaki, Yujiro Maeoka, Saori Nishio, Yasunori Utsunomiya, Shohei Nakanishi, Toshiki Doi, Shinsuke Harasawa, Takayasu Mori, Jun Ito, Kazuhiro Yoshikawa, Mitsuhiro Sato, Shoji Saito, Yoei Miyabe, Kentaro Nakai, Tomoaki Itoh, Hidaki Yoshida, Koji Sato, Hitoshi Suzuki, Tadashi Sofue, Masaaki Izumi, Yosuke Saka, Matsuhiko Hayashi, Seiji Kishi, Shu Ushimaru, Kaori Hayashi, Togo Aoyama, Keisuke Osaki, Hideki Fujii, Ryuji Iwatani, Shioko Okada, Kazufumi Nomura, Naomichi Uemura, Noriyuki Iehara, Mikoto Matsui, Tatsuo Tsukamoto, Tomomi Endo, Itaru Ebihara, Takayuki Hamano, Kohei Aoki, Yoshiharu Nishitani, Toshiyuki Imasawa, Masanori Abe, Masato Habuka, Yuko Shibuya, Keisuke Maruyama, Ken Shibuya, Chizuko Kitabayashi, Masami Komeda, Aya Nakamori, Jun Muratsu, Eiji Ishikawa, Norihisa Osawa, Ritsuko Hasegawa, Satoka Shiratori-Aso, Sachiko Takahashi, Hiroyuki Deguchi, Toshiyuki Aoki, Eiichi Sato, Shuzo Kobayashi, Hiroki Nishiwaki, Daisuke Ichikawa, Tomoko Yokoyama, Tetsuya Nagae, Takayuki Sakai, Shigeru Shibata, Jun Ino, Motokazu Matsuura, Rieko China, Tomonari Okada, Maiko Ohara, Hiroyuki Kinuno, Takayuki Toda, Ikuo Nukui, Jin Oshikawa, Makoto Ogi

**Affiliations:** 1 Department of Nephrology, Graduate School of Medicine, The University of Osaka, Suita, Osaka, Japan; 2 Department of Cardiology, Pulmonology, Hypertension & Nephrology, Ehime University Graduate School of Medicine, Toon, Ehime, Japan; 3 Health and Counseling Center, The University of Osaka, Toyonaka, Osaka, Japan; 4 Transdimensional Life Imaging Division, Institute for Open and Transdisciplinary Research Initiatives, The University of Osaka, Suita, Osaka, Japan; 5 Division of Nephrology and Endocrinology, The University of Tokyo Graduate School of Medicine, Tokyo, Japan

**Keywords:** Fanconi syndrome, nephrotoxicity

## Abstract

**Introduction:**

Dietary supplements are widely used and less strictly regulated than pharmaceuticals. Between 2023 and 2024, a nationwide outbreak of kidney injury associated with specific red yeast rice–containing supplements occurred in Japan, frequently presenting as acquired Fanconi syndrome. However, the subsequent clinical course of this condition remains unknown.

**Methods:**

This nationwide, physician-reported cohort study evaluated 190 adults with suspected supplement-associated kidney injury through the Japanese Society of Nephrology network. Longitudinal trajectories of estimated glomerular filtration rate (eGFR) and proximal tubular biochemical parameters were characterized using spline-based mixed-effects models. Kaplan-Meier methods estimated cumulative attainment probabilities for eGFR and biochemical thresholds.

**Results:**

Among 190 patients, 68% were female (median age category, 50–59 years); median eGFR at initial presentation was 29.2 ml/min per 1.73 m^2^ (interquartile range [IQR], 17.3–40.7). Modeled eGFR improved rapidly within the first 60 days after presentation but subsequently plateaued below 60 ml/min per 1.73 m^2^ over the follow-up period. Sensitivity analyses addressing differential follow-up yielded similar trajectories. Among patients with initial eGFR < 60 ml/min per 1.73 m^2^, the cumulative attainment of eGFR ≥ 60 ml/min per 1.73 m^2^ by day 540 was 33% (95% confidence interval [CI], 25%–44%). In contrast, Fanconi syndrome–related biochemical abnormalities, including hypokalemia, metabolic acidosis, hypouricemia, hypophosphatemia, glycosuria, and proteinuria, resolved rapidly, with ≥ 80% attaining target biochemical thresholds by day 120. Of 6 patients requiring acute dialysis, 1 progressed to permanent renal replacement therapy (RRT).

**Conclusion:**

Proximal tubular biochemical abnormalities largely resolved within 120 days, whereas eGFR plateaued early and often remained below 60 ml/min per 1.73 m^2^. This dissociation supports continued eGFR monitoring for potential chronic kidney disease despite biochemical resolution of proximal tubular dysfunction.

## Graphical abstract

**Figure:**
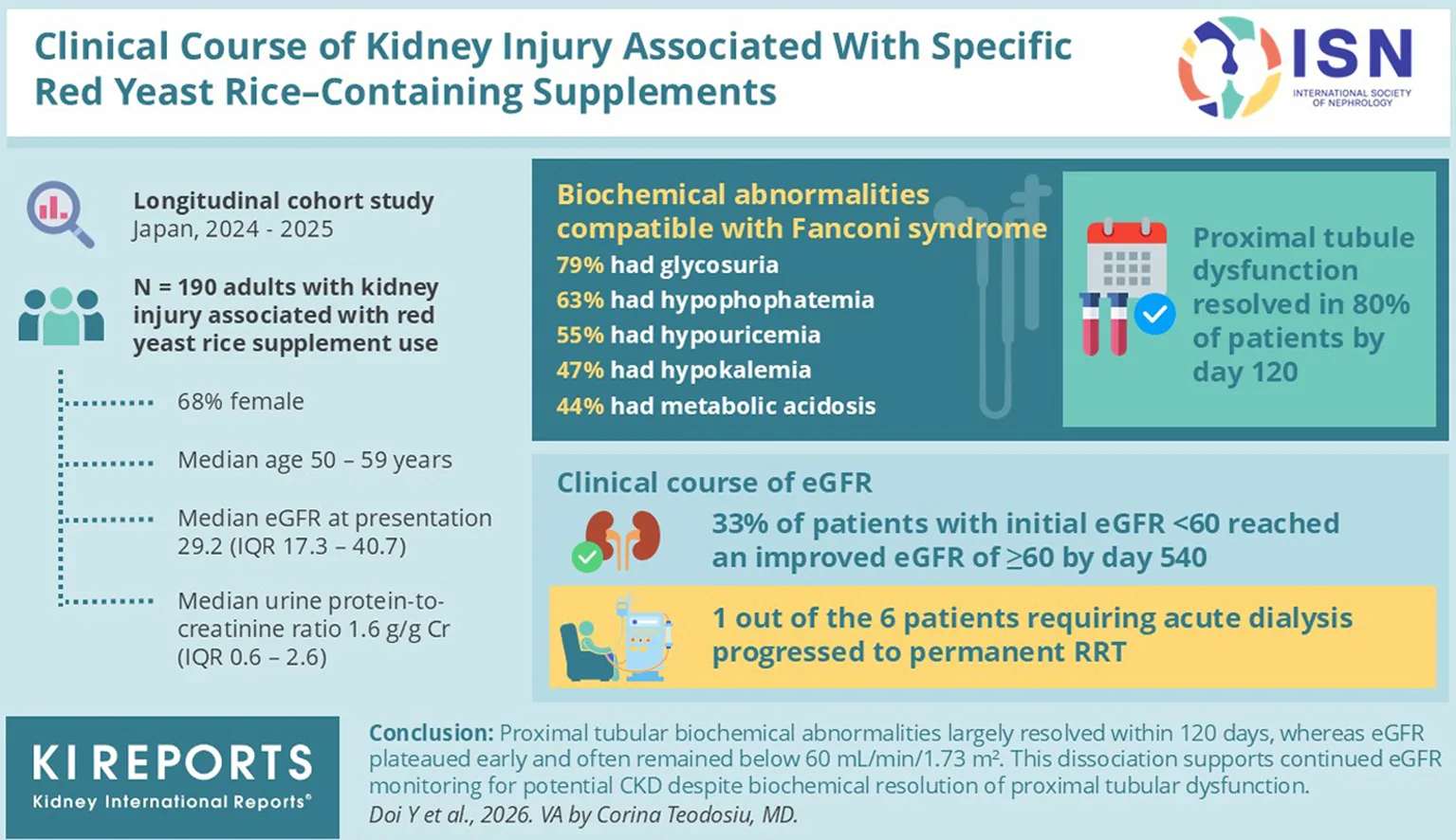

Dietary supplements are extensively consumed worldwide; however, they are often subject to less stringent premarket safety regulations and quality control standards than pharmaceuticals.^1^, ^2^ This regulatory gap poses a significant public health concern because consumers may inadvertently be exposed to unknown toxins or contaminated products.

Between late 2023 and early 2024, a cluster of kidney injury cases emerged in Japan among individuals who had consumed specific red yeast rice (Beni-koji) dietary supplements. Although puberulic acid was detected as a putative contaminant in implicated manufacturing lots,^3^, ^4^ its definitive pathogenic role remains under investigation.^5^, ^6^, ^7^ Initial reports from the Japanese Society of Nephrology identified a distinctive clinical pattern of proximal tubular dysfunction consistent with acquired Fanconi syndrome,^8^ often accompanied by significant reductions in eGFR. Notably, 87% of affected patients had an eGFR below 60 ml/min per 1.73 m^2^ at short-term follow-up.^9^

Because these supplements were marketed for cholesterol management, the affected population largely comprised middle-aged or older adults without a documented history of advanced chronic kidney disease. This cohort offers a unique opportunity to examine the clinical evolution of glomerular and tubular function following this specific type of toxin-associated kidney injury. Although the acute phase of this injury has been recently characterized, longitudinal data on the trajectories of both eGFR and tubular markers remain limited. To address this gap, we conducted a nationwide extension study through December 2025 to characterize longitudinal eGFR trajectories and the resolution patterns of biochemical abnormalities reflecting proximal tubular dysfunction.

## Methods

### Study Design and Participants

This nationwide, longitudinal cohort study is an extension of the investigation into kidney injury associated with specific red yeast rice supplements conducted by the Japanese Society of Nephrology. The project comprised 3 sequential phases using structured web-based questionnaires: the initial registration phase (phase 1: March 27–April 30, 2024), the short-term follow-up phase (phase 2: May 13–June 3, 2024), and the subsequent follow-up phase (phase 3: July 16–December 15, 2025). The first 2 phases have been previously described,^9^ and phase 3 is the focus of this report. Invitations for Phases 2 and 3 were restricted to physicians who responded to Phase 1, enabling the longitudinal evaluation of the initially identified cohort (Figure 1). All 3 phases were voluntary surveys directed to the attending physicians. Several reminder emails were sent to nonresponding physicians during phase 3; however, reasons for nonresponse were not systematically collected. The index date was defined as the date of the initial clinical visit reported by the attending physician in the phase 1 questionnaire.

**Figure 1:**
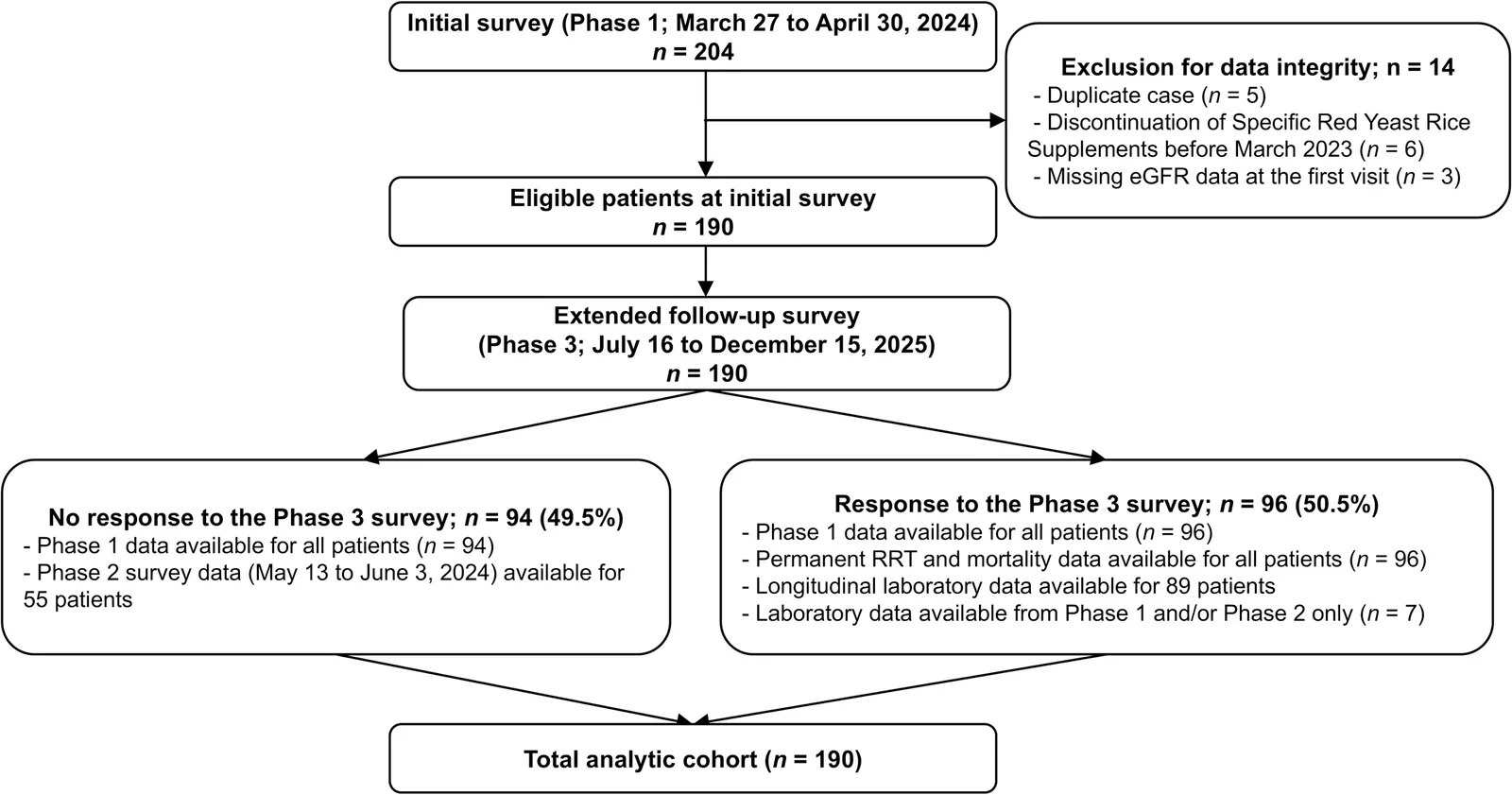
Study flow diagram. A total of 204 patients were initially registered in the phase 1 survey. After excluding duplicate cases (*n* = 5), patients who discontinued the specific supplements before March 2023 (*n* = 6), and those with missing eGFR data at the first visit (*n* = 3), 190 patients were included in the total analytic cohort. In the follow-up survey (phase 3), responses were obtained for 96 patients (50.5%), of whom 89 provided detailed longitudinal laboratory data. eGFR, estimated glomerular filtration rate; RRT, renal replacement therapy.

### Procedures

Consistent with the earlier phases, the attending physicians were requested to provide updated information regarding the date of the last visit, all-cause mortality, and the development of end-stage kidney disease necessitating permanent RRT. Detailed longitudinal clinical data were also retrieved from electronic medical records, encompassing blood and urine laboratory results from January 1, 2023, through the date of data extraction. The eGFR was calculated using the standardized Japanese equation: 194 × creatinine^-1.094^ × age^-0.287^ × (0.739 if female).^10^ Questionnaire items for each phase are detailed in Supplementary Tables S1–S3. This study was approved by the Ethics Committee of the University of Osaka Hospital (approval number 24010). Given the observational design, the requirement for written informed consent was waived, and an opt-out approach was implemented in accordance with national ethical guidelines for medical research involving human subjects.

### Statistical Analysis

Continuous variables were expressed as median (IQR), and categorical variables as numbers and percentages. To evaluate the time to attainment of eGFR thresholds, we used the Kaplan-Meier method to estimate the cumulative attainment probability of the first observed achievement of eGFR thresholds (≥ 45 or ≥ 60 ml/min per 1.73 m^2^). Among patients initially below these levels, attainment was defined as the first follow-up visit where the eGFR reached the target threshold. As a sensitivity analysis, we estimated the cumulative incidence of attaining eGFR ≥60 ml/min per 1.73 m^2^, treating death and permanent RRT as competing events. To analyze longitudinal eGFR trajectories, linear mixed-effects models with restricted cubic splines were employed.^11^ A 4-knot restricted cubic splines model—with knots at Harrell’s recommended percentiles (5th, 35th, 65th, and 95th)—was selected based on Akaike and Bayesian Information Criteria over cubic polynomial models. Trajectories were estimated up to 540 days (90th percentile of follow-up) to ensure data density. Models included fixed effects for spline terms of time, sex, and age category. To account for interindividual variability, we specified random intercepts and random linear slopes for time, using an unstructured covariance matrix to allow for correlation between initial levels and the rates of change. To assess the robustness of the estimated eGFR trajectory to differential availability of phase 3 longitudinal laboratory data, we performed an inverse probability-weighted sensitivity analysis based on observed characteristics at the initial visit. We also conducted scenario-based sensitivity analyses in which a hypothetical eGFR observation was added at day 360 for each patient without phase 3 longitudinal laboratory data, and the mixed-effects model was refitted under optimistic and pessimistic scenarios. Further details are provided in Supplementary Figures S1 and S2. As an additional sensitivity analysis, we evaluated the longitudinal eGFR trajectory in a subgroup restricted to patients without documented diabetes, hypertension, or preexisting kidney disease to minimize potential confounding from underlying comorbidities. Other continuous laboratory parameters were analyzed using similar mixed-effects models. Furthermore, to evaluate whether clinical characteristics at initial presentation, such as the presence of electrolyte abnormalities, modified the clinical course, interaction terms between these initial categories and the spline components of time were included. Urine protein-to-creatinine ratio (UPCR) values were log-transformed before modeling because of their skewed distribution. Mixed-effects models were fitted on the log scale, and predicted values were back-transformed to the original scale for visualization. Regarding qualitative urine glucose, longitudinal changes were evaluated using a mixed-effects ordinal logistic regression model with a similar fixed-effects structure and random intercepts. For visualization, marginal predicted probabilities for a representative patient profile (female, aged 50–59 years) were calculated and presented as stacked area plots. All statistical analyses were performed using Stata SE version 18.0 (StataCorp, College Station, TX).

### Patient and Public Engagement Summary

Patients or the public were not involved in the design, conduct, or reporting of this study.

## Results

### Study Population and Cohort Refinement

A total of 204 patients were identified through the initial survey (Figure 1). For the current follow-up study, we performed a rigorous data-cleaning process and excluded 14 patients: 5 duplicate cases, 6 patients who had discontinued the specific red yeast rice supplements before March 2023, and 3 patients without available eGFR data at the initial visit. Consequently, 190 unique patients were eligible for the final analytic cohort. This population represents a refined version of the 192 patients described in our previous report^9^; the slight difference in sample size stems from the removal of 4 additional duplicate cases and the inclusion of 2 patients whose initial laboratory data were newly retrieved during this follow-up study. The initial clinical and laboratory profiles of this refined cohort (*n* = 190) were highly consistent with our previous report, with no substantial differences observed in the prevalence or severity of kidney injury despite the minor change in the analytic population.

### Clinical Characteristics at Presentation

Characteristics at the initial clinical evaluation (Table 1) showed a female predominance (68%), with most patients in their 50s or 60s (73% combined). Preexisting comorbidities were uncommon, including hypertension (18%), kidney disease (9%), and diabetes (5%). At evaluation, median eGFR was 29.2 (IQR, 17.3–40.7) ml/min per 1.73 m^2^, with 52% of patients presenting below 30 ml/min per 1.73 m^2^. The median UPCR was 1.6 (IQR, 0.6–2.6) g/g Cr. Clinical hallmarks of Fanconi syndrome were highly prevalent, including hypokalemia (potassium < 3.5 mEq/l) in 47%, hypophosphatemia (phosphate < 2.5 mg/dl) in 63%, hypouricemia (uric acid < 2.0 mg/dl) in 55%, and metabolic acidosis (bicarbonate < 18 mmol/l) in 44%. Notably, in patients who underwent measurement of the tubular maximum reabsorption of phosphate per GFR (TmP/GFR)—who generally exhibited more severe electrolyte imbalances compared with those not measured (Supplementary Table S4)—the median TmP/GFR was 1.2 (IQR, 0.7–2.2) mg/dl. Collectively, these findings, together with the high prevalence of glycosuria (79%), are consistent with proximal tubular dysfunction.

**Table 1:** Clinical and laboratory characteristics of patients at initial presentation, stratified by eGFR (< 30 vs. ≥ 30 ml/min per 1.73 m^2^)

Characteristics	Missing, *n* (%)	Whole population	eGFR ≥ 30 ml/min per 1.73 m^2^	eGFR < 30 ml/min per 1.73 m^2^	*P*-value
		*N* = 190	*n* = 91	*n* = 99	
Demographics and comorbidities					
Age category, yr	0 (0%)				
30–39		5 (3%)	4 (4%)	1 (1%)	0.04
40–49		25 (13%)	14 (15%)	11 (11%)	
50–59		72 (38%)	39 (43%)	33 (33%)	
60–69		66 (35%)	29 (32%)	37 (37%)	
≥ 70		22 (12%)	5 (5%)	17 (17%)	
Female	0 (0%)	129 (68%)	61 (67%)	68 (69%)	0.81
History of kidney disease	62 (33%)	12 (9%)	4 (7%)	8 (11%)	0.41
Hypertension	60 (32%)	24 (18%)	8 (14%)	16 (22%)	0.25
Diabetes mellitus	59 (31%)	6 (5%)	2 (4%)	4 (5%)	0.61
Duration of specific RYR supplements use	2 (1%)				
≤ 3 mo		40 (21%)	19 (21%)	21 (21%)	0.58
4–7 mo		44 (23%)	24 (27%)	20 (20%)	
≥ 8 mo		104 (55%)	47 (52%)	57 (58%)	
Laboratory data					
Serum albumin (g/dl)	2 (1%)	4.2 (4.0–4.5)	4.2 (4.1–4.4)	4.2 (3.8–4.5)	0.49
eGFR (ml/min per 1.73 m^2^)	0 (0%)	29.2 (17.3–40.7)	41.8 (35.0–51.2)	17.6 (14.2–24.8)	< 0.01
Serum sodium (mEq/l)	1 (1%)	140 (138–142)	140 (139–142)	140 (137–141)	0.02
Serum potassium (mEq/l)	0 (0%)	3.5 (3.0–3.9)	3.6 (3.2–4.0)	3.4 (2.9–3.8)	0.14
Hypokalemia (serum potassium < 3.5 mEq/l)	0 (0%)	89 (47%)	38 (42%)	51 (52%)	0.18
Serum calcium (mg/dl)	9 (5%)	9.1 (8.8–9.5)	9.1 (8.6–9.5)	9.1 (8.8–9.5)	0.35
Serum phosphate (mg/dl)	11 (6%)	2.2 (1.7–3.0)	2.0 (1.4–2.9)	2.3 (1.8–3.1)	0.01
Hypophosphatemia (serum phosphate < 2.5 mg/dl)	11 (6%)	113 (63%)	58 (69%)	55 (58%)	0.12
TmP/GFR (mg/dl)	139 (73%)	1.2 (0.7–2.2)	1.2 (0.7–2.2)	1.3 (0.7–2.1)	0.91
Serum uric acid (mg/dl)	2 (1%)	1.8 (1.4–2.9)	1.8 (1.3–3.1)	1.9 (1.6–2.9)	0.24
Hypouricemia (serum uric acid < 2.0 mg/dl)	2 (1%)	103 (55%)	54 (60%)	49 (50%)	0.17
Bicarbonate (mmol/l)	44 (23%)	19.3 (15.7–23.7)	22.3 (19.3–25.2)	16.4 (13.5–20.3)	<0.01
Metabolic acidosis (bicarbonate < 18 mmol/l)	44 (23%)	64 (44%)	11 (17%)	53 (64%)	<0.01
UPCR (g/g Cr)	9 (5%)	1.6 (0.6–2.6)	0.8 (0.2–1.9)	2.2 (1.5–2.9)	<0.01
Urinary glucose (dipstick test)	2 (1%)				
Negative or trace		40 (21%)	33 (37%)	7 (7%)	< 0.01
1+		20 (11%)	14 (16%)	6 (6%)	
2+		16 (9%)	7 (8%)	9 (9%)	
3+		112 (60%)	36 (40%)	76 (78%)	

eGFR, estimated glomerular filtration rate; mo, month(s); RYR, Red Yeast Rice; TmP/GFR, tubular maximum phosphate reabsorption per glomerular filtration rate; UPCR, urinary protein-to-creatinine ratio, yr, year(s).

Values are presented as median (IQR) or *n* (%). *P*-values were calculated using the Wilcoxon rank-sum test for continuous variables and the chi-square test for categorical variables. Categorical variables with missing values were analyzed excluding the missing cases.

### Mortality and RRT

The previously reported fatality of a patient aged >70 years remains the sole death in this cohort. Of the 6 patients who initially required acute hemodialysis, only 1 transitioned to permanent RRT. Notably, this patient had preexisting kidney dysfunction (serum creatinine was 3.47 mg/dl, documented > 1 year prior) and comorbid hydronephrosis, which likely contributed to the irreversibility of their kidney failure. No additional deaths or new RRT initiations were identified in the phase 3 observation survey.

### Longitudinal eGFR Trajectories

The median follow-up for the total cohort was 133 days (IQR, 60–481). The median follow-up was 483 days (IQR, 434–531) for patients with laboratory data obtained through the phase 3 survey (*n* = 89) versus 67 days (IQR, 29–102) for those without (*n* = 101). Patients with phase 3 longitudinal laboratory data had lower eGFR and serum potassium levels and a higher prevalence of eGFR < 30 ml/min per 1.73 m^2^ at initial presentation than those without such data (Supplementary Table S5). Among patients with an eGFR < 60 ml/min per 1.73 m^2^ at the initial visit, eGFR ≥ 60 ml/min per 1.73 m^2^ was observed in 14/69 (20.3%), 10/65 (15.4%), and 2/26 (7.7%) patients around days 180, 365, and 540, respectively (Supplementary Table S6). Mixed-effects model analysis estimated a rapid initial recovery of eGFR during the first 60 days after the initial visit. This improvement subsequently slowed and reached a plateau, with eGFR levels remaining stable through day 540 (representing the 90th percentile of follow-up) (Figure 2a). The inverse probability-weighted sensitivity analysis for differential availability of phase 3 longitudinal laboratory data yielded an eGFR trajectory similar to the unweighted reference trajectory (Supplementary Figure S1). Furthermore, in scenario-based sensitivity analyses, we added a hypothetical day 360 eGFR observation for each patient without phase 3 longitudinal laboratory data. In the optimistic scenario, the hypothetical value was set at 15 ml/min per 1.73 m^2^ above the reference-model prediction (68.0 vs. 53.0 ml/min per 1.73 m^2^), and the refitted trajectory showed no further improvement beyond the early phase. In the pessimistic scenario, the value was set at 15 ml/min per 1.73 m^2^ below the reference-model prediction (38.0 vs. 53.0 ml/min per 1.73 m^2^), and the refitted trajectory showed a gradual decline after the initial improvement (Supplementary Figure S2). A sensitivity analysis restricting the cohort to 92 patients without documented diabetes, hypertension, or preexisting kidney disease yielded consistent trajectories (Supplementary Figure S3).

**Figure 2:**
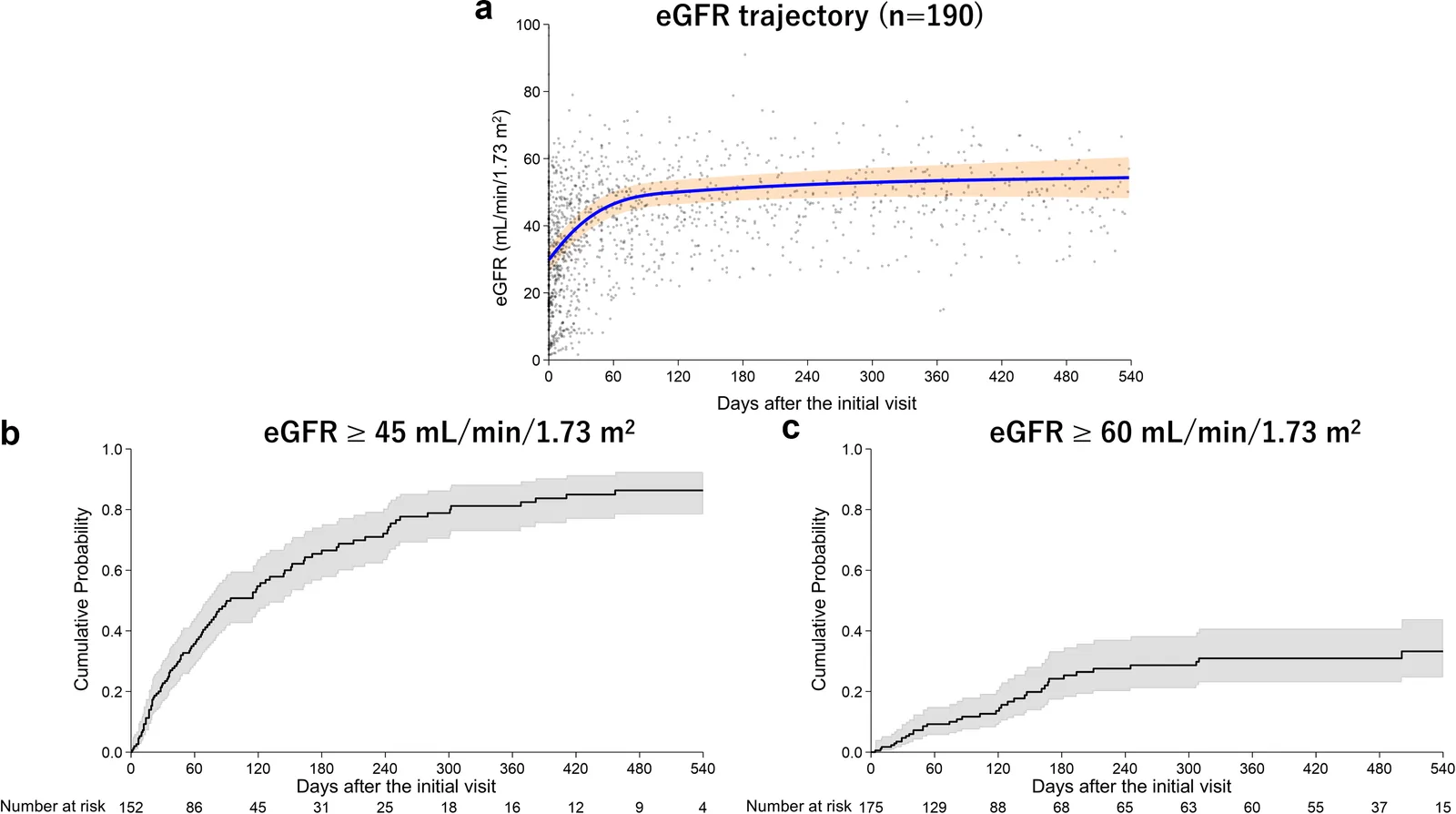
Longitudinal eGFR trajectories and attainment of eGFR thresholds. (a) Predicted and observed eGFR trajectories during the 540-day follow-up period (90th percentile of follow-up). The solid blue line represents the predicted eGFR values estimated using a linear mixed-effects model with a 4-knot restricted cubic spline to account for nonlinear trends. The model was based on 1488 observations from 190 patients, corresponding to a mean of 7.8 measurements per patient and a median of 4 (interquartile range, 3–12; range, 1–30). Knots were positioned at the 5th, 35th, 65th, and 95th percentiles (0, 17, 117, and 480 days, respectively). The light orange shaded area indicates the 95% CI, and gray dots represent individual observed measurements. (b, c) Time to first attainment of eGFR ≥ 45 (b) and ≥ 60 (c) ml/min per 1.73 m^2^. Kaplan-Meier curves (solid black lines) with 95% CIs (gray shaded areas) illustrate the cumulative attainment probability for each eGFR threshold. The numbers of participants at risk for each time point are displayed below the horizontal axis. CI, confidence interval; eGFR, estimated glomerular filtration rate.

### Time to Reach eGFR ≥ 45 or ≥ 60 ml/min per 1.73 m^2^

Kaplan-Meier analysis estimated the time to reach specific eGFR thresholds. In patients with initial eGFR < 45 ml/min per 1.73 m^2^, the median time to achieve eGFR **≥** 45 was 94 days (95% CI, 72–144), with a cumulative attainment of 86.3% (95% CI, 78.4–92.5) at 540 days (Figure 2b). Conversely, for the **≥** 60 ml/min per 1.73 m^2^ threshold among those with initial values below this level, the cumulative attainment at 540 days was only 33% (95% CI, 25–44; Figure 2c and Supplementary Table S7). After excluding the patient receiving permanent RRT at the index visit and treating the single death during follow-up as a competing event, the cumulative incidence of attaining eGFR ≥ 60 ml/min per 1.73 m^2^ at day 540 was 33% (95% CI, 24%–43%).

### Trajectory of Fanconi syndrome–related Parameters

Regarding serum laboratory parameters, observed medians at initial presentation for potassium, bicarbonate, uric acid, and phosphate were 3.5 mEq/l, 19.3 mmol/l, 1.8 mg/dl, and 2.2 mg/dl, respectively. Based on the linear mixed-effects models, these parameters were estimated to reach clinical reference ranges rapidly within 60 to 120 days of the initial visit (Figure 3a–d). Consistent with these model-based trends, Kaplan-Meier analysis confirmed substantial clinical recovery during the same period, with more than 80% of patients achieving targets by day 120 (Supplementary Table S7). Urinary biomarkers also showed comparable improvement; the predicted geometric mean of UPCR declined to approximately 0.1 g/g Cr by day 60 (Figure 3e). Although the mixed-effects model estimated a nearly immediate recovery in the probability of negative or trace glycosuria (Figure 3f), Kaplan-Meier analysis indicated a slightly more gradual clinical recovery, though over 80% of patients reached the target thresholds by day 120 (Supplementary Table S7). In the subset measured (*n* = 51), the observed median TmP/GFR at initial evaluation was 1.2 mg/dl, which was estimated to rise to approximately 3.0 mg/dl by day 120 (Figure 3g).

**Figure 3:**
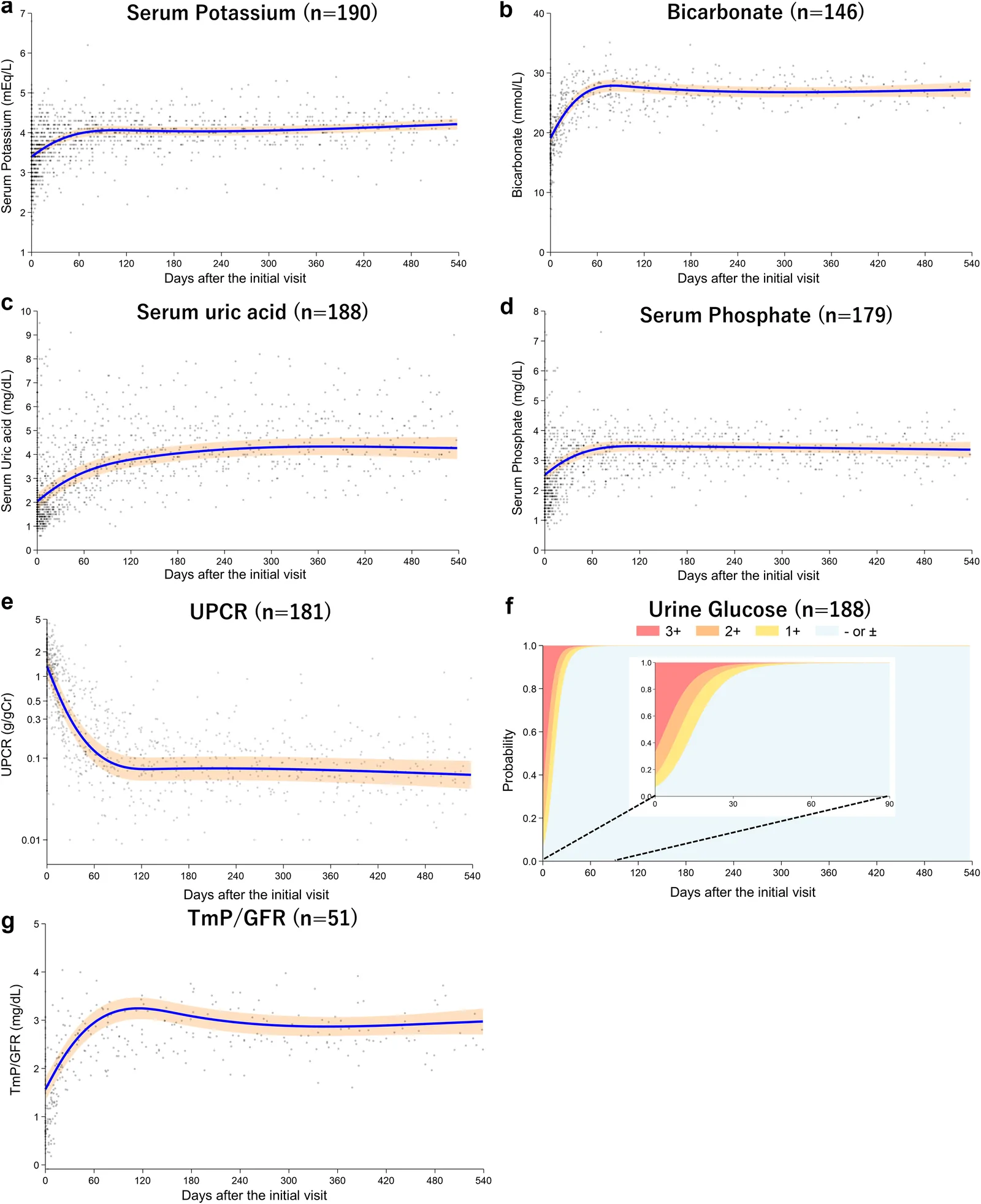
Longitudinal trajectories of parameters related to Fanconi syndrome. (a–d) Serum parameters. Predicted trajectories (solid blue lines) with 95% CIs (shaded areas) are shown for (a) potassium (*n* = 190), (b) bicarbonate (*n* = 146), (c) uric acid (*n* = 188), and (d) phosphate (*n* = 179). These markers showed rapid improvement toward reference ranges within 60–120 days. (e) UPCR (*n* = 181). Predicted geometric means (solid lines) with 95% CIs (shaded areas) estimated on a log scale. (f) Urine glucose (*n* = 188). Marginal predicted probabilities of urine glucose severity categories estimated using a mixed-effects ordered logistic regression model. The main plot illustrates the full 540-day period, whereas the inset provides a magnified view of the first 90 days to highlight the rapid recovery phase. (g) TmP/GFR (*n* = 51). Predicted longitudinal trajectory (solid lines) with 95% CIs (shaded areas) in the measured subset. CI, confidence interval; TmP/GFR, Tubular maximum phosphate reabsorption per glomerular filtration rate; UPCR, urinary protein-to-creatinine ratio.

### Subgroup Analysis

Subgroup analysis estimated a significant group-by-time interaction for eGFR trajectories based on initial eGFR levels (< 30 vs. ≥ 30 ml/min per 1.73 m^2^; P for interaction < 0.01; Figure 4a). Patients in the initial eGFR < 30 ml/min per 1.73 m^2^ group exhibited a more rapid estimated initial increase; however, their eGFR levels reached a lower plateau and remained consistently lower than those of the higher initial eGFR subgroup throughout the follow-up period. In contrast, the duration of supplement intake did not significantly modify these trajectories (P for interaction = 0.66; Supplementary Figure S4). Analyses of other Fanconi syndrome–related parameters—stratified by the presence of observed abnormalities at presentation, including hypokalemia (potassium < 3.5 mEq/l), metabolic acidosis (bicarbonate < 18 mmol/l), hypouricemia (uric acid < 2.0 mg/dl), and hypophosphatemia (phosphate < 2.5 mg/dl)—also demonstrated significant heterogeneity in their longitudinal trajectories (P for interaction < 0.01). In each case, the group with observed abnormalities at presentation exhibited a more rapid and pronounced recovery (Figure 4b–e). Similarly, for UPCR, the group with an initial UPCR > 0.3 g/g Cr demonstrated a rapid estimated decline and achieved normalization during the follow-up period (Figure 4f).

**Figure 4:**
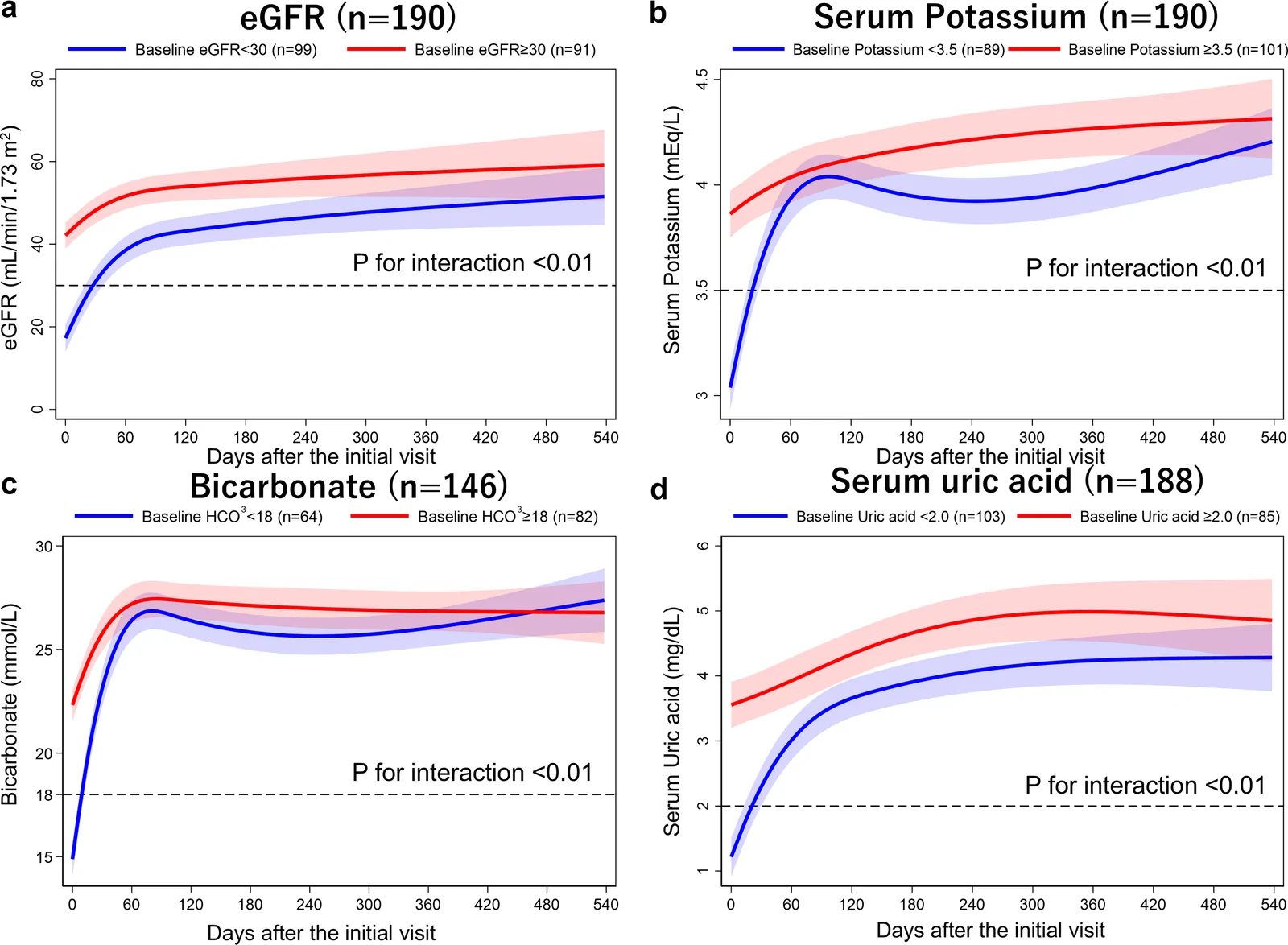
Subgroup analyses stratified by severity at initial presentation. (a) eGFR trajectories. Data are stratified by initial eGFR levels (< 30 vs. ≥ 30 ml/min per 1.73 m^2^). Solid lines and shaded areas represent predicted trajectories and 95% CIs, respectively. (b–e) Serum parameters. Predicted trajectories (solid lines) with 95% CIs (shaded areas) for (b) potassium (*n* = 190), (c) bicarbonate (*n* = 146), (d) uric acid (*n* = 188), and (e) phosphate (*n* = 179), stratified by the presence of abnormalities at presentation. (f) UPCR (*n* = 181). Data are stratified by initial UPCR levels (> 0.3 vs. ≤ 0.3 g/g Cr). Solid lines and shaded areas represent predicted trajectories and 95% CIs, respectively. Significant group-by-time interactions (*P* < 0.01) were observed for all parameters, with the groups exhibiting initial abnormalities showing more pronounced initial improvement. CI, confidence interval; UPCR, urinary protein-to-creatinine ratio.

## Discussion

In this nationwide longitudinal study, we characterized the clinical course and medium-term kidney outcomes of a large-scale public health incident involving kidney injury associated with contaminated red yeast rice supplements. Although earlier reports established the acute phenotype as acquired Fanconi syndrome, the present study provides longitudinal evidence on recovery trajectories extending to 540 days. The primary finding is that the modeled eGFR trajectory showed substantial initial improvement after supplement cessation but subsequently plateaued below 60 ml/min per 1.73 m^2^ during the observed follow-up period. This longitudinal characterization moves beyond the initial diagnosis and suggests that kidney function may remain impaired in some affected patients despite biochemical resolution of proximal tubular dysfunction.

To our knowledge, this represents one of the largest longitudinal analyses of a mass-exposure nephrotoxic event linked to a single dietary supplement. Although other incidents, most notably aristolochic acid nephropathy, represent a vast and ongoing global public health crisis,^12^, ^13^ granular longitudinal data in such cohorts remain limited. Our analysis reveals that although eGFR initially improved after discontinuing the supplements, the estimated cohort-level eGFR trajectory plateaued below 60 ml/min per 1.73 m^2^. Notably, progression to end-stage kidney disease was rare—a sharp contrast to the typically relentless and progressive clinical course of aristolochic acid nephropathy.^13^, ^14^ This relatively favorable outcome may partially reflect the truncated exposure; puberulic acid was detected only in batches shipped after July 2023, and the public health warning in March 2024 effectively limited the duration of ingestion.^15^ Although the current study defines the eGFR trajectory up to 540 days, extended monitoring remains imperative to elucidate the lifetime prognosis. Reflecting the experience with aristolochic acid nephropathy, prolonged surveillance is warranted to confirm kidney functional stability and screen for late-onset extra-kidney sequelae, including malignancies.^12^

Although the improvement in tubular markers followed a timeframe similar to that of eGFR, the functional recovery profiles were distinct: reabsorptive defects frequently returned to laboratory reference ranges. The longitudinal tracking of glycosuria resolution and TmP/GFR changes provides insights into the clinical course of this condition. Subgroup analyses demonstrated that patients with abnormalities at initial presentation, including hypokalemia, metabolic acidosis, hypouricemia, and hypophosphatemia, exhibited substantial improvement toward reference ranges (Figure 4b–e). Similarly, UPCR in patients with elevated initial levels decreased rapidly during follow-up (Figure 4f). This resolution of reabsorptive defects is consistent with the hypothesized mechanism of injury. Recent evidence has implicated puberulic acid as a putative contaminant that impairs mitochondrial bioenergetics,^5^, ^16^ including reported effects on cytochrome c oxidase.^17^ Because the proximal tubule is the most metabolically active segment of the nephron, it possesses a significant capacity to restore function if mitochondrial inhibition is attenuated, a process reflective of the intrinsic regenerative potential of the tubular epithelium.^8^, ^18^ However, the mechanisms underlying the persistent eGFR reduction remain uncertain. Maladaptive repair after acute kidney injury, including interstitial fibrosis and nephron loss, represents a biologically plausible explanation,^19^ but these mechanisms could not be evaluated in the present study. Thus, the observed dissociation indicates that resolution of proximal tubular biochemical abnormalities did not necessarily parallel recovery of overall filtration capacity.

This crisis demonstrates the real-world impact of global regulatory challenges in the dietary supplement market.^1^ The fact that the initial signal was identified by astute clinicians underscores the need for a robust and integrated toxicovigilance network to complement existing frameworks. Ultimately, the medical community must remain vigilant; when encountering unexplained tubular dysfunction or Fanconi syndrome, a thorough inquiry into dietary supplement history is paramount. Such clinical alertness serves as an important component of toxicovigilance, particularly for over-the-counter supplements, which are frequently omitted from patient-reported medication histories.^20^

A strength of this study is the availability of detailed longitudinal data, including follow-up extending to 540 days, in a nationwide cohort of 190 patients, enabling characterization of medium-term recovery trajectories. The involvement of the Japanese Society of Nephrology facilitated standardized data collection during a national crisis and enabled detailed characterization of both glomerular and proximal tubular recovery after kidney injury associated with these specific supplements. Several limitations must be acknowledged. First, selection may have occurred at the initial physician-reporting stage; milder, unrecognized, non–nephrology-managed, or otherwise unreported cases may be underrepresented, and this cohort cannot define population incidence or the full severity spectrum. In addition, as a questionnaire-based survey, the study is subject to reporting and recall biases, and some laboratory data were unavailable at specific time points. Second, the absence of preexposure eGFR data precludes a definitive assessment of whether the observed eGFR plateau represents a return to premorbid function or persistent impairment. The overall eGFR status in this cohort was notably lower than age-specific Japanese population norms (where the prevalence of reduced eGFR is only 5%–20% for ages 50–69),^21^ suggesting possible residual kidney injury beyond age-related decline. Third, the phase 3 survey response rate was approximately 50%. This nonresponse may have introduced selection bias because patients with more severe or persistent kidney impairment may have remained under clinical follow-up, whereas those who recovered rapidly may have been discharged. Therefore, censoring in the Kaplan-Meier analyses may have been informative, potentially biasing the estimated cumulative attainment probabilities. Although inverse probability-weighted and scenario-based sensitivity analyses yielded findings consistent with the primary analysis, these analyses cannot fully resolve uncertainty arising from potentially informative missingness.

In conclusion, our nationwide survey of patients with kidney injury associated with specific red yeast rice supplements demonstrated a distinct clinical course: biochemical abnormalities reflecting proximal tubular dysfunction largely resolved during follow-up, whereas the estimated cohort-level eGFR trajectory improved initially and subsequently plateaued below 60 ml/min per 1.73 m^2^. Unexplained organ dysfunction, especially involving the kidneys, should immediately trigger a thorough investigation of dietary supplement use. Clinicians must maintain a high suspicion for over-the-counter products and ensure that suspected toxicities are reported promptly. Combined with sustained follow-up, such professional alertness serves as a critical early-warning system to detect unrecognized health hazards and protect the public.

## Appendix

### List of the Collaborators

Masami Ono (Ageo Central General Hospital); Yuriko Yonekura (Akashi Medical Center); Yuki Nishizawa (Anzu Clinic Asakusabashi); Yuri Kasagi (Association of EISEIKAI Medical and Healthcare Corporation Minamitama Hospital); Masahiro Azuma (Azuma Kidney Clinic); Ryousuke Aoki (Chiba Tokushukai Hospital); Koji Inagaki (Chutoen General Medical Center); Keiji Kono (Division of Nephrology and Kidney Center, Kobe University Graduate School of Medicine); Akihiro Tojo (Dokkyo Medical University); Hideyuki Katori (Ebina General Hospital); Ken-ichi Miyoshi (Ehime University Graduate School of Medicine); Satoru Shichijo (Ehime University Graduate School of Medicine); Akiko Torii (Eiju General Hospital); Reina Miyazaki (Fuji City General Hospital); Sinsuke Motegi (Fujioka General Hospital); Yasutaka Kamikawa (Fukui-ken Saiseikai Hospital); Emi Kitashoji (FUKUOKA HIGASHI HOBASHIRA CLINIC); Takehiko Tokura (Fukuyama City Hospital); Yoshikazu Nemoto (Gengendo Kimitsu Hospital); Masahito Baba (Gunma Saiseikai Maebashi Hospital); Risshi Kudo (Hamanasu Clinic); Atsushi Saito (Harunohi Medical Clinic); Ryuta Fujimura (Higashiosaka City Medical Center); Takashi Araki (Hino Municipal Hospital); Hiraku Yoshida (Hiraku Clinic); Takanori Miyazaki (Hiratsuka Kyosai Hospital); Daiki Nagawa (Hirosaki University Graduate School of Medicine); Yosuke Osaki (Hiroshima University Hospital); Yujiro Maeoka (Hiroshima University Hospital); Saori Nishio (Hokkaido University Hospital); Yasunori Utsunomiya (Hoya Clinic); Shohei Nakanishi (Hyogo Prefectural Harima-Himeji General Medical Center); Toshiki Doi (Ichiyokai Harada Hospital); Shinsuke Harasawa (Iidabashi West-Gate Clinic); Takayasu Mori (Institute of Science Tokyo); Jun Ito (International University of Health and Welfare); Kazuhiro Yoshikawa (Iwate Medical University); Mitsuhiro Sato (Japan Community Healthcare Organization Sendai Hospital); Shoji Saito (Japanese Red Cross Aichi Medical Center Nagoya Daini Hospital); Yoei Miyabe (Japanese Red Cross Kumamoto Hospital); Kentaro Nakai (Japanese Red Cross Fukuoka Hospital); Tomoaki Itoh (JCHO Saitama Medical Center); Hidaki Yoshida (JR Sapporo Hospital); Koji Sato (Juntendo Nerima Hospital); Hitoshi Suzuki (Juntendo University Urayasu Hospital); Tadashi Sofue (Kagawa University); Masaaki Izumi (Kansai Rosai Hospital); Yosuke Saka (Kasugai Municipal Hospital); Matsuhiko Hayashi (KAWAKITA GENERAL HOSPITAL); Seiji Kishi (Kawasaki Medical School); Shu Ushimaru (Kawasaki Municipal Tama Hospital); Kaori Hayashi (Keio University School of Medicine); Togo Aoyama (Kitasato University Hospital); Keisuke Osaki (Kobe City Medical Center General Hospital); Hideki Fujii (Kobe University Graduate School of Medicine); Ryuji Iwatani (Koga Hospital 21); Shioko Okada (Konan Medical Center); Kazufumi Nomura (Kumamoto Chuo Hospital); Naomichi Uemura (Kumamoto General Hospital); Noriyuki Iehara (Kyoto City Hospital); Mikoto Matsui (Matsui Heart Clinic); Tatsuo Tsukamoto (Medical Research Institute KITANO HOSPITAL); Tomomi Endo (Medical Research Institute KITANO HOSPITAL); Itaru Ebihara (Mito Saiseikai General Hospital); Takayuki Hamano (Nagoya City University Graduate School of Medical Sciences); Kohei Aoki (Nakatsu Municipal Hospital); Yoshiharu Nishitani (Nara City Hospital); Toshiyuki Imasawa (NHO Chibahigashi National Hospital); Masanori Abe (Nihon University School of Medicine); Masato Habuka (Niigata Prefectural Shibata Hospital); Yuko Shibuya (NTT Medical Center Tokyo); Keisuke Maruyama (Okayama Saiseikai General Hospital); Ken Shibuya (Omori Red Cross Hospital); Chizuko Kitabayashi (Osaka City General Hospital); Masami Komeda (Osaka Pref. Saiseikai Tondabayashi Hospital); Aya Nakamori (Otemae Hospital); Jun Muratsu (Rinku General Medical Center); Eiji Ishikawa (Saiseikai General Hospital Department of Nephrology); Norihisa Osawa (Saiseikai Shiga Hospital); Ritsuko Hasegawa (Sanai Memorial Soga Clinic); Satoka Shiratori-Aso (Sapporo City General Hospital); Sachiko Takahashi (Seiwa Clinic); Hiroyuki Deguchi (Sendai-Makinose Urology-Nephrology Clinic); Toshiyuki Aoki (Shimbashi Aoki Clinic); Eiichi Sato (Shinmatsudo Central General Hospital); Shuzo Kobayashi (Shonan Kamakura General Hospital); Hiroki Nishiwaki (Showa Medical University Fujigaoka Hospital); Daisuke Ichikawa (St. Marianna University School of Medicine); Tomoko Yokoyama (Takamatsu Red Cross Hospital); Tetsuya Nagae (Takashima Municipal Hospital); Takayuki Sakai (Takeru Clinic); Shigeru Shibata (Teikyo University School of Medicine); Jun Ino (Toda Central General Hospital); Motokazu Matsuura (Tokushima Red Cross Hospital); Rieko China (Tokyo Medical University Hospital); Tomonari Okada (Tokyo Metropolitan Police Hospital); Maiko Ohara (Toyama Machinaka Hospital); Hiroyuki Kinuno (Toyama Rosai Hospital); Takayuki Toda (Tsuchiura Kyodo General Hospital); Ikuo Nukui (Yamanashi Prefectural Central Hospital); Jin Oshikawa (Yokohama Sakae Kyosai Hospital); Makoto Ogi (Yuurinkouseikai Fuji Hospital).

## Disclosure

All authors declared no competing interests.

## Supplementary Material

Supplementary File (PDF) **Figure S1.** Sensitivity analysis for the longitudinal eGFR trajectory using inverse probability weighting. **Figure S2.** Scenario-based sensitivity analysis for the longitudinal eGFR trajectory using hypothetical day 360 observations**. Figure S3.** Longitudinal eGFR trajectory in patients without documented diabetes, hypertension, or preexisting kidney disease. **Figure S4.** Longitudinal eGFR trajectories stratified by duration of supplement intake. **Table S1.** Contents of the initial phase questionnaire (phase 1; survey period: March 27–April 30, 2024). **Table S2.** Contents of the follow-up phase questionnaire (phase 2; survey period: May 13 – June 3, 2024). **Table S3.** Contents of the extended follow-up questionnaire (phase 3; survey period: July 16–December 15, 2025). **Table S4.** Characteristics at initial presentation, stratified by the measurement of TmP/GFR. **Table S5.** Characteristics at initial presentation, stratified by the availability of follow-up data from the phase 3 survey. **Table S6.** Observed eGFR at selected follow-up landmarks among patients with an eGFR <60 ml/min per 1.73 m^2^ at the initial visit. **Table S7.** Kaplan-Meier estimates of cumulative attainment of eGFR and tubular biochemical targets. STROBE Checklist

## Footnotes

Supplementary File (PDF)
**Figure S1.** Sensitivity analysis for the longitudinal eGFR trajectory using inverse probability weighting. **Figure S2.** Scenario-based sensitivity analysis for the longitudinal eGFR trajectory using hypothetical day 360 observations. **Figure S3.** Longitudinal eGFR trajectory in patients without documented diabetes, hypertension, or preexisting kidney disease. **Figure S4.** Longitudinal eGFR trajectories stratified by duration of supplement intake. **Table S1.** Contents of the initial phase questionnaire (phase 1; survey period: March 27–April 30, 2024). **Table S2.** Contents of the follow-up phase questionnaire (phase 2; survey period: May 13 – June 3, 2024). **Table S3.** Contents of the extended follow-up questionnaire (phase 3; survey period: July 16 – December 15, 2025). **Table S4.** Characteristics at initial presentation, stratified by the measurement of TmP/GFR. **Table S5.** Characteristics at initial presentation, stratified by the availability of follow-up data from the phase 3 survey. **Table S6.** Observed eGFR at selected follow-up landmarks among patients with an eGFR <60 ml/min per 1.73 m^2^ at the initial visit. **Table S7.** Kaplan-Meier estimates of cumulative attainment of eGFR and tubular biochemical targets. STROBE Checklist.
