# Supplementary material for: Clinical Course of Kidney Injury Associated With Specific Red Yeast Rice–Containing Supplements
Source: Kidney Int Rep. 2026 Sep 30;11(12):107107. doi: 10.1016/j.ekir.2026.107107 (PMC13733332; doi:10.1016/j.ekir.2026.107107)
Supplement: Supplementary file 1 [file mmc1.pdf]

Table of Contents for the Supplemental Materials

Supplementary Table S1.

Contents of the initial phase questionnaire (Phase 1; Survey period: March 27 – April 30, 2024)

Supplementary Table S2.

Contents of the follow-up phase questionnaire (Phase 2; Survey period: May 13 – June 3, 2024)

Supplementary Table S3.

Contents of the extended follow-up questionnaire (Phase 3; Survey period: July 16 – December 15, 2025)

Supplementary Table S4.

Characteristics at Initial Presentation, Stratified by the Measurement of TmP/GFR

Supplementary Table S5.

Characteristics at Initial Presentation, Stratified by the Availability of Follow-up Data from the Phase 3 Survey

Supplementary Table S6.

Observed eGFR at Selected Follow-up Landmarks Among Patients With an eGFR <60 mL/min/1.73 m^2 at the Initial Visit

Supplementary Table S7.

Kaplan–Meier Estimates of Cumulative Attainment of eGFR and Tubular Biochemical Targets

Supplementary Figure S1.

Sensitivity Analysis for the Longitudinal eGFR Trajectory Using Inverse Probability Weighting

Supplementary Figure S2.

Scenario-Based Sensitivity Analysis for the Longitudinal eGFR Trajectory Using Hypothetical Day 360 Observations

Supplementary Figure S3.

Longitudinal eGFR Trajectory in Patients Without Documented Diabetes, Hypertension, or Pre-existing Kidney Disease

Supplementary Figure S4.

Longitudinal eGFR Trajectories Stratified by Duration of Supplement Intake

Supplementary Table S1. Contents of the initial phase questionnaire (Phase 1; Survey period: March 27 – April 30, 2024)

	Questionnaire items
1.	E-mail address
2.	Name
3.	Affiliation
4.	Patient's age
	- ~19 years
	- 20–29 years
	- 30–39 years
	- 40–49 years
	- 50–59 years
	- 60–69 years
	- 70+ years
5.	Patient's gender
	- Male
	- Female
6.	Which Beni-koji-containing product brand names were used?
	(Multiple selections possible)
	- Beni-koji Choleste-Help®
	- Naishi-Help Plus Cholesterol®
	- Natto-Kinase SaraSara granules GOLD®
	- Other (specify)
7.	When did the patient start taking Beni-koji Choleste-Help® or similar products?
	(Options from before March 2023 to March 2024)
Questions Related to Initial Consultation:	
8.	When was the initial consultation related to this issue?
	(Options from before March 2023 to March 2024)
9.	What were the chief complaints? (Multiple selections possible)
	- Fever (37°C or higher)
	- Fatigue
	- Loss of appetite
	- Weight loss
	- Weight gain
	- Edema

- Urinary abnormalities
- Renal dysfunction
- Abdominal symptoms
- Skin rash
- Joint pain
- Other (specify)

Questions Related to Initial or Referral Examination Data:	
10.	White blood cell count? /μL
11.	Eosinophil count? %
12.	Serum sodium (Na) level? mEq/L
13.	Serum potassium (K) level? mEq/L
14.	Blood urea nitrogen (BUN) level? mg/dL
15.	Serum uric acid (UA) level? mg/dL
16.	Serum creatinine (Cre) level? mg/dL
17.	Estimated glomerular filtration rate (eGFR)? mL/min/1.73m^2
18.	Serum calcium (Ca) level? mg/dL
19.	Serum phosphorus (P) level? mg/dL
20.	Serum creatine kinase (CK) level? IU/L
21.	Serum albumin (Alb) level? g/dL
22.	Serum CRP level? mg/dL
23.	Bicarbonate ion (HCO_3^-) level? mEq/L
24.	Urinary protein (qualitative)?<br>- negative or trace<br>- 1+<br>- 2+<br>- 3+ or more
25.	Urinary protein (quantitative)? g/gCr
26.	Urinary occult blood (qualitative)?<br>- negative or trace<br>- 1+<br>- 2+<br>- 3+ or more
27.	Urinary glucose (qualitative)?<br>- negative or trace<br>- 1+<br>- 2+

- 3+ or more

28. Urinary uric acid (UA) level? mg/dL
29. Urinary creatinine (Cre) level? mg/dL
30. Urinary inorganic phosphorus (P) level? mg/dL
31. Urinary β2-microglobulin (β2MG) level? ng/mL (mg/L)
32. Urinary N-acetyl-β-D-glucosaminidase (NAG) level? IU/L

Questions about renal biopsy:

---

33. Was a renal biopsy performed?
    - No
    - Yes (If yes, answer the following questions)
34. When was the renal biopsy performed?
    (Options from before March 2023 to March 2024)
35. Light microscopy findings? (Multiple selections possible)
    - Minor glomerular abnormalities
    - Focal segmental glomerulosclerosis
    - Membranous nephropathy
    - Membranoproliferative glomerulonephritis
    - Endocapillary proliferative glomerulonephritis
    - Crescentic glomerulonephritis
    - Mesangial proliferative glomerulonephritis
    - Tubulointerstitial nephritis
    - Tubular necrosis
    - Thrombotic microangiopathy
    - Microscopic polyangiitis
    - Diabetic nephropathy
    - Other (specify)
36. Was electron microscopy performed?
    - No
    - Yes (If yes, answer the following question)
37. Electron microscopy findings?

Questions about treatment:

---

38. When was Beni-koji Choleste-Help® or similar product discontinued?
    (Options from "Not discontinued" to March 2024)
39. Was glucocorticoid used?
    - Yes
    - No

40.	Was dialysis performed?<br>- Yes<br>- No
41.	Other treatments? If any, please describe below.
Questions about outcome:	
42.	Maximum creatinine (Cre) value during the course? mg/dL
43.	When was the maximum creatinine (Cre) value observed?
44.	Date of the most recent serum creatinine (Cre) test?
45.	Most recent serum creatinine (Cre) value? mg/dL
46.	Most recent estimated glomerular filtration rate (eGFR) value? mL/min/1.73m^2
47.	How did the electrolyte abnormalities change?<br>- Electrolyte abnormalities improved<br>- Electrolyte abnormalities remained unchanged<br>- Electrolyte abnormalities worsened<br>- Other (specify)
48.	How did the urinary findings change?<br>- Urinary abnormalities improved<br>- Urinary abnormalities remained unchanged<br>- Urinary abnormalities worsened<br>- Other (specify)
49.	Did the patient die?<br>- Yes<br>- No
Questions about the causal relationship with Beni-koji Choleste-Help®:	
50.	How do you assess the causal relationship between this case and Beni-koji Choleste-Help® or similar products?<br>- Unknown<br>- Possible<br>- Probable<br>- Definitive
Other	
51.	If you have any additional comments about this case, please describe below.

Note: Table S1 is adapted from the previous study (Kidney International. 2025; 107: 530–540). The e-mail address, name, and affiliation items refer to the responding physician.

Supplementary Table S2. Contents of the follow-up phase questionnaire (Phase 2; Survey period: May 13 – June 3, 2024)

	Questionnaire items
1.	Email address
2.	Name
3.	Affiliation
4.	Preference for inclusion in acknowledgments
	- None
	- Yes (If yes, please provide hospital name and name in English below)
5.	Name (English)
6.	Affiliation (English)
7.	Patient's age
	[Same as in Phase 1]
8.	Patient's gender
	[Same as in Phase 1]
9.	History of kidney disease? (Multiple selections possible)
	- Unknown history
	- No history
	- Chronic glomerulonephritis
	- Rapidly progressive glomerulonephritis
	- Nephrosclerosis
	- Diabetic nephropathy
	- Interstitial nephritis
	- Polycystic kidney disease
	- Unknown primary disease
	- Other (specify)
10.	History of hypertension?
	- Yes
	- No
	- Unknown
11.	History of diabetes?
	- Yes
	- No
	- Unknown
12.	Which Beni-koji related products were used? (Multiple selections possible)

[Same as in Phase 1]

13. When did the patient start taking Beni-koji Choleste-Help or similar products?

    [Same as in Phase 1]

14. When was the initial consultation related to this issue?

    [Same as in Phase 1]

15. What were the chief complaints? (Multiple selections possible)

    [Same options as in Phase 1]

16-38. Laboratory values

    [Same as in Phase 1]

39-44. Renal biopsy questions

    [Similar to Phase 1, with additional question on electron microscopy findings other than tubular damage]

45-51. Treatment and outcome questions

    [Similar to Phase 1, with additional question on discontinuation of dialysis]

Additional survey items:

52-68. Most recent laboratory values (if tests were performed between the previous survey and May 31, 2024)

69. How do you assess the causal relationship between this case and Beni-koji Choleste-Help or similar products?

    [Same as in Phase 1]

70. If you have any additional comments about this case, please describe below

Note: Table S2 is adapted from the previous study (Kidney International. 2025; 107: 530–540). The e-mail address, name, and affiliation items refer to the responding physician.

Supplementary Table S3. Contents of extended follow-up questionnaire (Phase 3; Survey period: July 16 – December 15, 2025)

	Questionnaire items
1.	Email address
2.	Name (Previous contact person)
3.	Affiliation (Previous contact person)
4.	Name (Current person in charge)
5.	Affiliation (Current person in charge)
6.	Preference for inclusion in acknowledgments
	- None
	- Yes (If yes, please provide hospital name and name in English below)
7.	Name (English)
8.	Affiliation (English)
For confirmation	
9.	Patient's age
	[Same as in Phase 1]
10.	Patient's gender
	[Same as in Phase 1]
11.	When did the patient start taking Beni-koji Choleste-Help or similar products?
	[Same as in Phase 1]
12.	When was the initial consultation related to this issue?
	[Same as in Phase 1]
13.	What were the chief complaints? (Multiple selections possible)
	[Same options as in Phase 1]
14.	When was Beni-koji Choleste-Help® or similar product discontinued?
	[Same options as in Phase 1]
Additional information about patient characteristics and prognosis	
15.	Date of the Patient's birthday?
16.	Date of the patient's last visit?
17.	Did the patient require permanent renal replacement therapy (RRT) for end-stage kidney disease (ESKD)?
	- No
	- Yes (If yes, answer the following questions)
18.	Date of RRT initiation?
19.	Did the patient die?

- No
- Yes (If yes, answer the following questions)

20.	Date of death?
Please upload the following longitudinal data in Excel format. (Target period: January 1, 2023, to the date of data extraction)	
21.	Data of blood and urine laboratory test results
22.	Data of prescription and medication order information<br>(including oral medications and infusions)
Other	
23.	If you have any additional comments about this case, please describe below.

Note: The e-mail address, name, and affiliation items refer to the responding physician.

Supplementary Table S4. Characteristics at Initial Presentation, Stratified by the Measurement of TmP/GFR

	Missing, n (%)	Whole population	Without TmP/GFR	With TmP/GFR	p-value
		N=190	N=139	N=51	
**Demographics and Comorbidities**					
**Age category, years**	0 (0%)				
**30–39**		5 (3%)	4 (3%)	1 (2%)	0.08
**40–49**		25 (13%)	14 (10%)	11 (22%)	
**50–59**		72 (38%)	49 (35%)	23 (45%)	
**60–69**		66 (35%)	53 (38%)	13 (25%)	
**≥ 70**		22 (12%)	19 (14%)	3 (6%)	
**Female**	0 (0%)	129 (68%)	94 (68%)	35 (69%)	0.90
**Duration of Specific RYR Supplements use**	2 (1%)				
**≤ 3 months**		40 (21%)	27 (20%)	13 (26%)	0.30
**4–7 months**		44 (23%)	36 (26%)	8 (16%)	
**≥ 8 months**		104 (55%)	75 (54%)	29 (58%)	
**Laboratory data**					
**Serum albumin (g/dL)**	2 (1%)	4.2 (4.0–4.5)	4.2 (4.0–4.5)	4.2 (3.8–4.4)	0.28
**eGFR (mL/min/1.73 m^2)**	0 (0%)	29.2 (17.3–40.7)	29.5 (16.8–42.0)	29.1 (18.7–38.8)	0.98
**eGFR < 30 mL/min/1.73 m^2**	0 (0%)	99 (52%)	71 (51%)	28 (55%)	0.64
**Serum sodium (mEq/L)**	1 (1%)	140 (138–142)	140 (138–142)	140 (138–141)	0.45
**Serum potassium (mEq/L)**	0 (0%)	3.5 (3.0–3.9)	3.6 (3.1–4.0)	3.3 (2.8–3.6)	<0.01

**Hypokalemia (serum potassium < 3.5 mEq/L)**	0 (0%)	89 (47%)	54 (39%)	35 (69%)	<0.01
**Serum calcium (mg/dL)**	9 (5%)	9.1 (8.8–9.5)	9.1 (8.8–9.6)	9.1 (8.7–9.4)	0.51
**Serum phosphate (mg/dL)**	11 (6%)	2.2 (1.7–3.0)	2.3 (1.7–3.1)	1.9 (1.4–2.4)	0.01
**Hypophosphatemia (serum phosphate < 2.5 mg/dL)**	11 (6%)	113 (63%)	74 (58%)	39 (76%)	0.02
**Serum uric acid (mg/dL)**	2 (1%)	1.8 (1.4–2.9)	1.9 (1.4–3.4)	1.7 (1.5–2.3)	0.11
**Hypouricemia (serum uric acid < 2.0 mg/dL)**	2 (1%)	103 (55%)	69 (50%)	34 (67%)	0.05
**Bicarbonate (mmol/L)**	44 (23%)	19.3 (15.7–23.7)	19.4 (15.5–23.2)	18.8 (15.8–24.1)	0.97
**Metabolic acidosis (bicarbonate < 18 mmol/L)**	44 (23%)	64 (44%)	44 (45%)	20 (42%)	0.71
**UPCR (g/gCr)**	9 (5%)	1.6 (0.6–2.6)	1.6 (0.5–2.5)	1.9 (1.1–2.9)	0.08
**Urinary glucose (dipstick test)**	2 (1%)				0.52
**Negative or trace**		40 (21%)	32 (23%)	8 (16%)	
**1+**		20 (11%)	16 (12%)	4 (8%)	
**2+**		16 (9%)	11 (8%)	5 (10%)	
**3+**		112 (60%)	78 (57%)	34 (67%)	

Note: Values are presented as median (IQR) or n (%). p-values were calculated using the Wilcoxon rank-sum test for continuous variables and the chi-square test for categorical variables. Categorical variables with missing values were analyzed excluding the missing cases.

Abbreviations: TmP/GFR, Tubular maximum phosphate reabsorption per glomerular filtration rate; RYR, Red Yeast Rice; eGFR, estimated glomerular filtration rate; UPCR, urinary protein-to-creatinine ratio.

Supplementary Table S5. Characteristics at Initial Presentation, Stratified by the Availability of Follow-up Data from the Phase 3 Survey

	Missing, n (%)	Whole population	Without Phase 3 laboratory data	With Phase 3 laboratory data	p-value
		N=190	N=101	N=89	
**Demographics and Comorbidities**					
**Age category, years**	0 (0%)				0.19
**30–39**		5 (3%)	3 (3%)	2 (2%)	
**40–49**		25 (13%)	13 (13%)	12 (13%)	
**50–59**		72 (38%)	35 (35%)	37 (42%)	
**60–69**		66 (35%)	33 (33%)	33 (37%)	
**≥ 70**		22 (12%)	17 (17%)	5 (6%)	
**Female**	0 (0%)	129 (68%)	70 (69%)	59 (66%)	0.66
**Duration of Specific RYR Supplements use**	2 (1%)				
**≤ 3 months**		40 (21%)	17 (17%)	23 (26%)	0.28
**4–7 months**		44 (23%)	26 (26%)	18 (20%)	
**≥ 8 months**		104 (55%)	57 (57%)	47 (53%)	
**Laboratory data**					
**Serum albumin (g/dL)**	2 (1%)	4.2 (4.0–4.5)	4.3 (4.1–4.5)	4.1 (3.8–4.4)	0.05
**eGFR (mL/min/1.73 m^2)**	0 (0%)	29.2 (17.3–40.7)	31.0 (17.6–42.9)	27.0 (17.1–37.2)	0.08
**eGFR < 30 mL/min/1.73 m^2**	0 (0%)	99 (52%)	43 (43%)	56 (63%)	<0.01
**Serum sodium (mEq/L)**	1 (1%)	140 (138–142)	140 (138–142)	140.0 (138–141)	0.36
**Serum potassium (mEq/L)**	0 (0%)	3.5 (3.0–3.9)	3.6 (3.2–4.0)	3.4 (2.9–3.7)	<0.01

**Hypokalemia (serum potassium < 3.5 mEq/L)**	0 (0%)	89 (47%)	38 (38%)	51 (57%)	<0.01
**Serum calcium (mg/dL)**	9 (5%)	9.1 (8.8–9.5)	9.1 (8.8–9.6)	9.0 (8.7–9.4)	0.27
**Serum phosphate (mg/dL)**	11 (6%)	2.2 (1.7–3.0)	2.2 (1.6–3.1)	2.1 (1.7–2.9)	0.64
**TmP/GFR (mg/dL)**	139 (73%)	1.2 (0.7–2.2)	NA	1.2 (0.7–2.2)	NA
**Hypophosphatemia (serum phosphate < 2.5 mg/dL)**	11 (6%)	113 (63%)	55 (54%)	58 (65%)	0.09
**Serum uric acid (mg/dL)**	2 (1%)	1.8 (1.4–2.9)	2.0 (1.3–3.6)	1.8 (1.5–2.4)	0.27
**Hypouricemia (serum uric acid < 2.0 mg/dL)**	2 (1%)	103 (55%)	48 (48%)	55 (62%)	0.08
**Bicarbonate (mmol/L)**	44 (23%)	19.3 (15.7–23.7)	19.4 (15.4–23.7)	19.3 (15.7–23.1)	0.96
**Metabolic acidosis (bicarbonate < 18 mmol/L)**	44 (23%)	64 (44%)	30 (30%)	34 (38%)	0.01
**UPCR (g/gCr)**	9 (5%)	1.6 (0.6–2.6)	1.2 (0.3–2.4)	2.0 (1.1–2.8)	<0.01
**Urinary glucose (dipstick test)**	2 (1%)				0.29
**Negative or trace**		40 (21%)	26 (26%)	14 (16%)	
**1+**		20 (11%)	13 (13%)	7 (8%)	
**2+**		16 (9%)	8 (8%)	8 (9%)	
**3+**		112 (60%)	53 (52%)	59 (66%)	

Note: Values are presented as median (IQR) or n (%). p-values were calculated using the Wilcoxon rank-sum test for continuous variables and the chi-square test for categorical variables. Categorical variables with missing values were analyzed excluding the missing cases.

Abbreviations: RYR, Red Yeast Rice; eGFR, estimated glomerular filtration rate; TmP/GFR, Tubular maximum phosphate reabsorption per glomerular filtration rate; UPCR, urinary protein-to-creatinine ratio.

Supplementary Table S6. Observed eGFR at Selected Follow-up Landmarks Among Patients With an eGFR <60 mL/min/1.73 m² at the Initial Visit

Parameter	Day 180	Day 365	Day 540
Landmark window, days	150–210	335–395	510–570
Patients with an available eGFR measurement, n	69	65	26
Observed eGFR, median [IQR], mL/min/1.73 m²	47.0 [41.4–54.0]	49.6 [44.0–57.3]	50.0 [43.6–53.0]
Observed eGFR ≥60 mL/min/1.73 m², n/N (%)	14/69 (20.3%)	10/65 (15.4%)	2/26 (7.7%)

Note: Values are presented as median [interquartile range] or n/N (%). Analyses were restricted to patients with an eGFR <60 mL/min/1.73 m² at the initial visit. For each landmark, patients with at least one eGFR measurement within ±30 days of the target day were included, and the measurement closest to the target day was selected. When two measurements were equally distant from the target day, the earlier measurement was selected. No imputation or model-based estimation was used. Because different patients contributed measurements at each landmark and measurement availability may have been related to clinical status, these summaries should not be interpreted as cumulative attainment probabilities or as longitudinal changes within a fixed cohort.

Supplementary Table S7. Kaplan–Meier Estimates of Cumulative Attainment of eGFR and Tubular Biochemical Targets

Parameter (Cut-off)	Abnormality at Initial Presentation, n/N (%)	Analyzed in KM (n)	Day 60 (%) [95% CI]	Day 120 (%) [95% CI]	Day 180 (%) [95% CI]	Day 360 (%) [95% CI]	Day 540 (%) [95% CI]
**Glomerular Function**							
eGFR (≥ 60 mL/min/1.73 m²)	183/190 (96%)	175	9 [6–15]	14 [9–21]	24 [17–33]	31 [23–41]	33 [25–44]
**Tubular Function: Blood Markers**							
Serum potassium (≥ 3.5 mEq/L)	89/190 (47%)	69	68 [57–79]	88 [80–95]	96 [89–99]	96 [89–99]	—
Bicarbonate (≥ 18 mmol/L)	64/146 (44%)	40	68 [53–81]	93 [82–98]	100 [—]	—	—
Serum uric acid (≥ 2.0 mg/dL)	103/188 (55%)	75	63 [52–74]	84 [75–92]	98 [91–100]	98 [91–100]	—
Serum phosphate (≥ 2.5 mg/dL)	113/179 (63%)	79	66 [56–76]	87 [78–93]	95 [87–98]	98 [92–100]	—
**Tubular Function: Urinary Markers**							
UPCR (< 0.3 g/gCr)	151/181 (83%)	111	51 [41–60]	80 [78–88]	96 [91–99]	96 [91–99]	—
Urinary glucose (≤ trace)	148/188 (79%)	106	53 [44–63]	83 [75–89]	96 [91–99]	96 [91–99]	—

Note: Cumulative attainment probabilities were estimated using the Kaplan–Meier method. For each parameter, the analysis was restricted to patients who did not meet the specified target at the initial visit. "Analyzed in KM (n)" represents patients who did not meet the target at the initial visit and had at least one follow-up measurement. The "—" symbol indicates that estimates are not shown at later time points because cumulative attainment had plateaued near 100% or the number of patients remaining at risk was insufficient for reliable estimation.

Abbreviations: eGFR, estimated glomerular filtration rate; KM, Kaplan–Meier; UPCR, urine protein-to-creatinine ratio.

Supplementary Figure S1. Sensitivity Analysis for the Longitudinal eGFR Trajectory Using Inverse Probability Weighting.

The solid black line represents the reference analysis using the same 4-knot restricted cubic spline specification as the primary analysis, refitted in the sensitivity-analysis cohort (n = 188, excluding the two patients who died or initiated permanent RRT). To assess potential selection bias associated with the availability of Phase 3 longitudinal laboratory data, we performed an inverse probability-weighted sensitivity analysis. The probability of having Phase 3 longitudinal laboratory data was estimated using a logistic regression model incorporating sex, age category, and eGFR, serum potassium, serum uric acid, and semiquantitative urinary glucose at the initial visit. Stabilized patient-level weights, truncated at the 1st and 99th percentiles, were applied to the mixed-effects model among patients with Phase 3 longitudinal laboratory data and complete data for the variables included in the weighting model (n = 86). The blue dashed line represents the weighted eGFR trajectory. Its close agreement with the reference trajectory indicates that adjustment for these observed differences at the initial visit did not materially alter the estimated trajectory. The horizontal gray dashed line indicates an eGFR of 60 mL/min/1.73 m². Marginal predicted values are presented for a representative patient profile (female, aged 50–59 years).

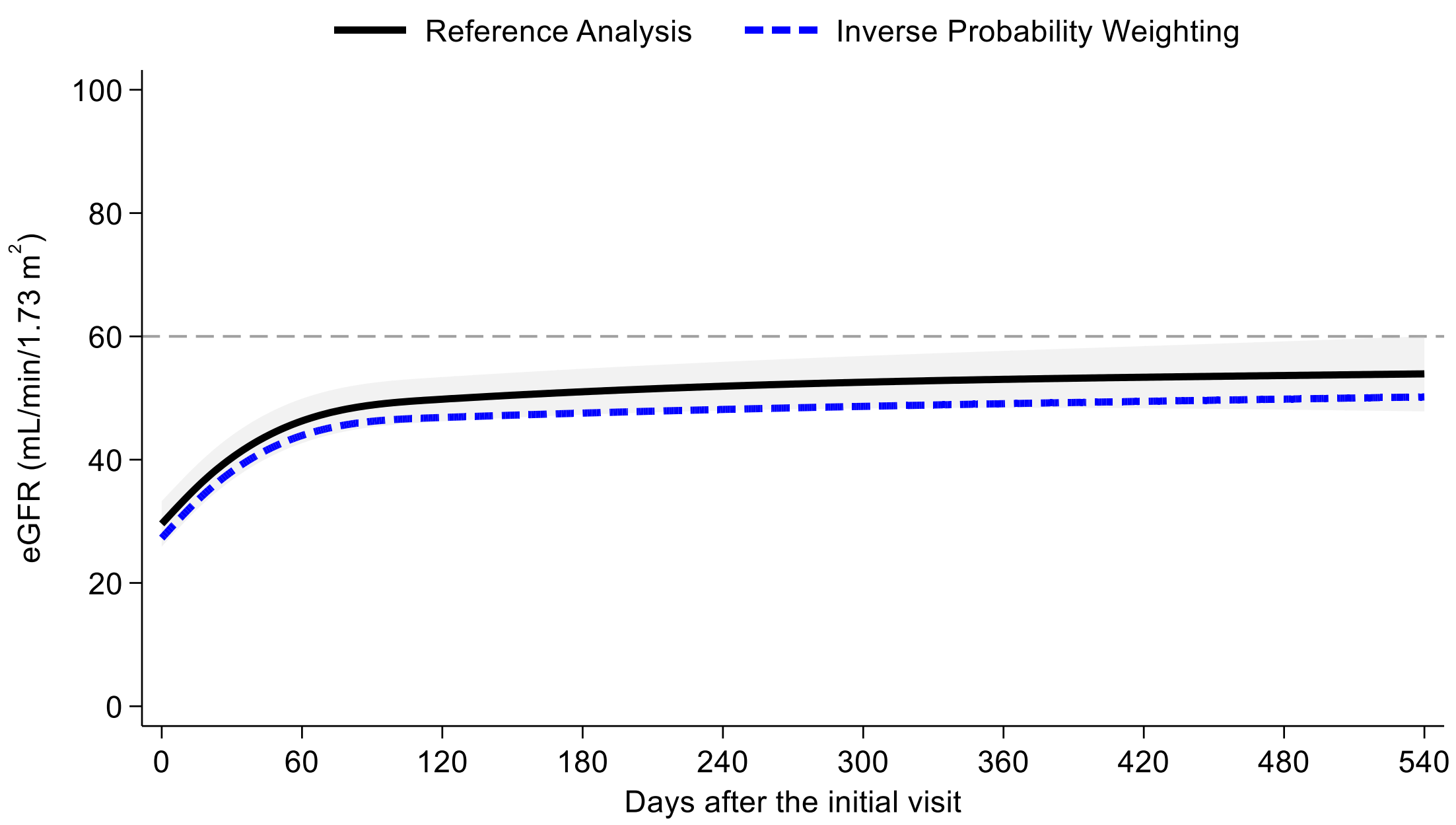

Supplementary Figure S2. Scenario-Based Sensitivity Analysis for the Longitudinal eGFR Trajectory Using Hypothetical Day 360 Observations

The solid black line represents the reference analysis using the same 4-knot restricted cubic spline specification as the primary analysis, refitted in the sensitivity-analysis cohort (n = 188, excluding the two patients who died or initiated permanent RRT). To examine whether patients without Phase 3 longitudinal laboratory data might have had systematically different kidney function, we performed scenario-based sensitivity analyses. For each of these patients, one hypothetical eGFR observation was added at Day 360, and the mixed-effects model was refitted under each scenario.

Because no empirically established value was available for this clinical setting, the hypothetical eGFR was set at 15 mL/min/1.73 m² above or below the Day 360 marginal prediction from the reference model. Day 360 was selected as a clinically interpretable approximately 1-year landmark within the plateau phase observed after Day 120. The reference-model estimate at Day 360 was approximately 53 mL/min/1.73 m². Accordingly, the hypothetical value was set to approximately 68 mL/min/1.73 m² in the optimistic scenario and 38 mL/min/1.73 m² in the pessimistic scenario. These three values corresponded to KDIGO GFR categories G2, G3a, and G3b, respectively.

The blue dashed and red short-dashed lines represent the refitted trajectories under the optimistic and pessimistic scenarios, respectively. The horizontal gray dashed line indicates an eGFR of 60 mL/min/1.73 m². Marginal predicted values are presented for a representative patient profile (female, aged 50–59 years).

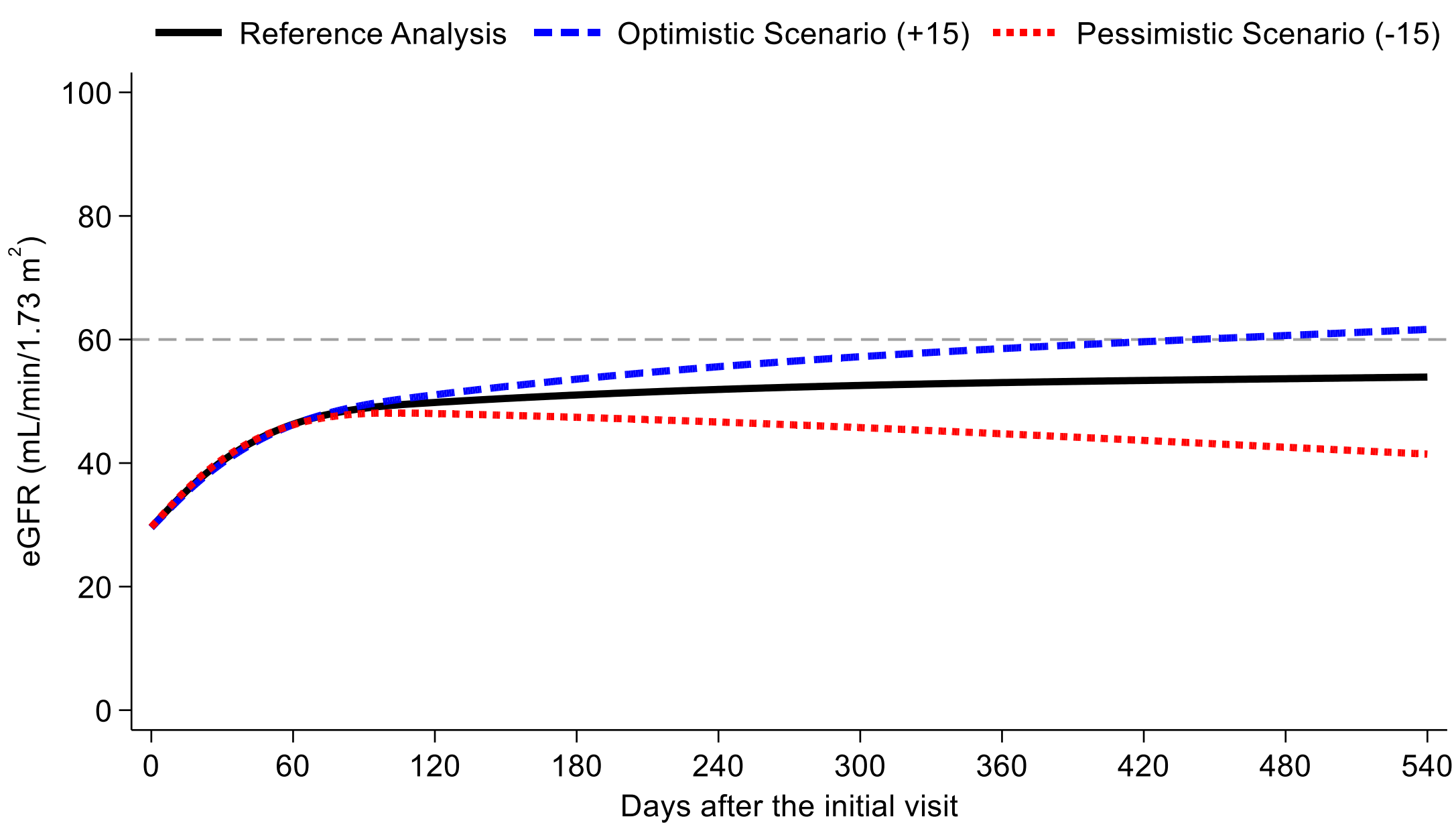

Supplementary Figure S3. Longitudinal eGFR Trajectory in Patients Without Documented Diabetes, Hypertension, or Pre-existing Kidney Disease.

The estimated trajectory demonstrates a rapid initial recovery of eGFR followed by a plateau below 60 mL/min/1.73 m², closely consistent with the primary analysis in the whole cohort. Trajectories were estimated using a linear mixed-effects 4-knot restricted cubic spline model adjusted for age category and sex. The analysis was restricted to 92 patients with documented absence of diabetes, hypertension, and pre-existing kidney disease. The blue solid line represents the marginal predicted eGFR trajectory, and the shaded area indicates the corresponding 95% confidence interval. Gray dots represent individual observed eGFR measurements. Marginal predicted values are presented for a representative patient profile (female, aged 50–59 years).

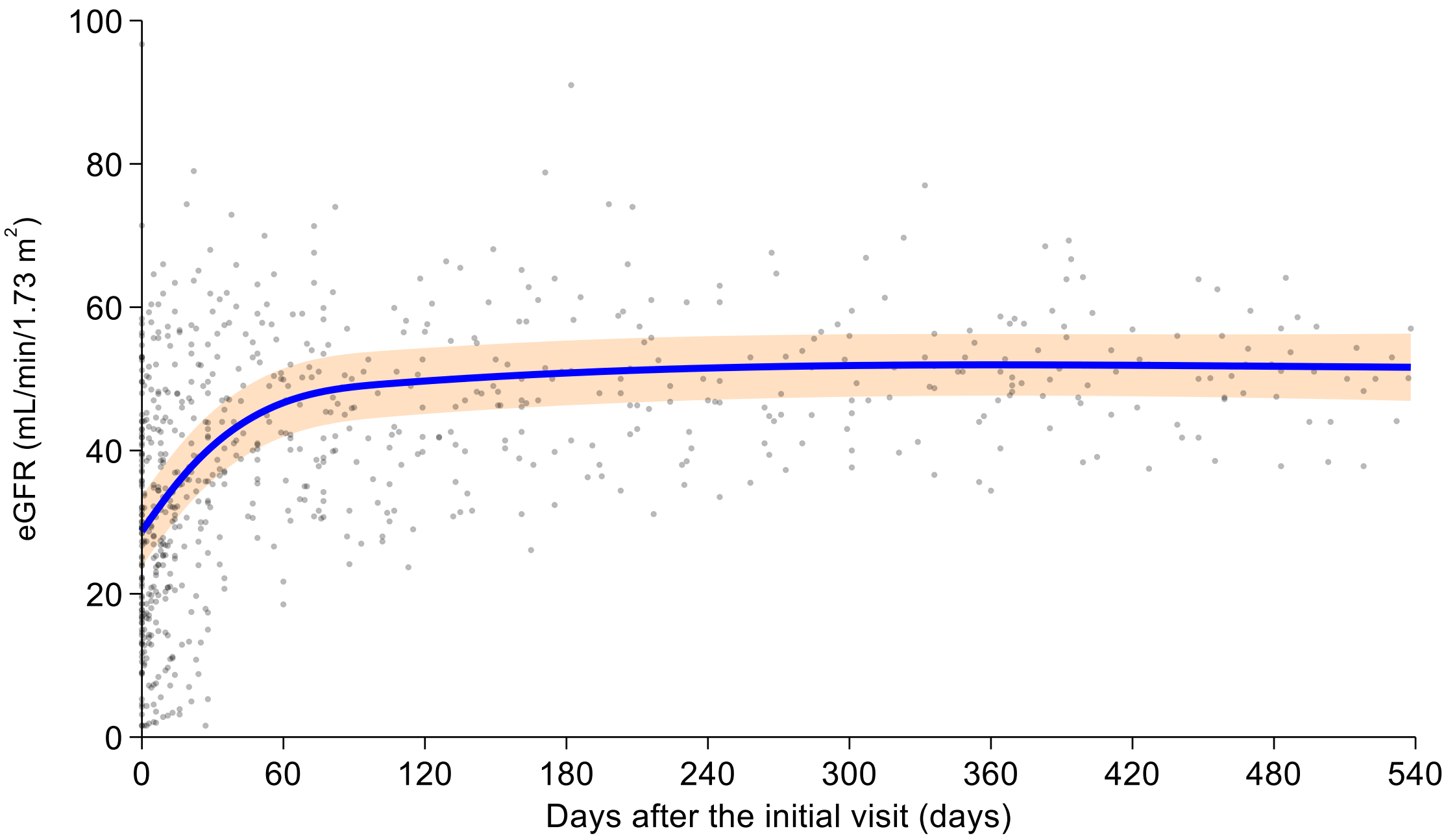

Supplementary Figure S4. Longitudinal eGFR Trajectories Stratified by Duration of Supplement Intake.

The predicted trajectories were largely overlapping, and the interaction between intake duration categories and the spline components of time was not significant (P for interaction = 0.66). Trajectories were estimated using a mixed-effects 4-knot restricted cubic spline model including interaction terms between intake duration category and the spline components of time, adjusted for age category and sex. Patients were categorized based on the duration of supplement intake: ≤3 months (blue solid line), 4–7 months (red dashed line), and ≥8 months (green dotted line). The shaded areas represent the corresponding 95% confidence intervals. The horizontal gray dashed line indicates the eGFR threshold of 60 mL/min/1.73 m². Marginal predicted values are presented for a representative patient profile (female, aged 50–59 years).

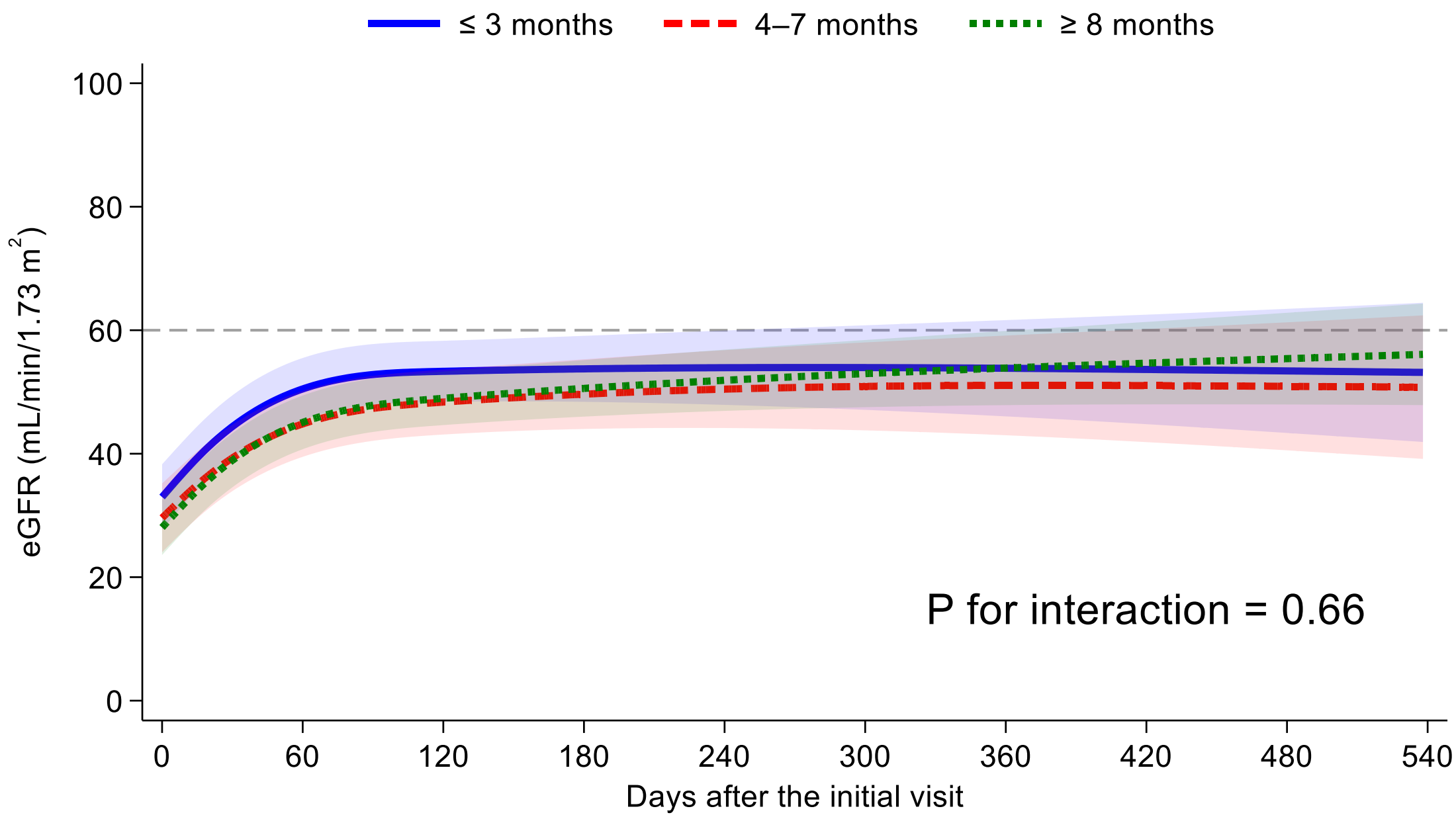

STROBE Statement—Checklist of Items for Cohort Studies

Page-only version

Section	Item No.	Recommendation	Page(s)
Title and abstract	1(a)	Indicate the study's design with a commonly used term in the title or the abstract	Pages 1
	1(b)	Provide in the abstract an informative and balanced summary of what was done and what was found	Pages 3-4
Introduction	2	Explain the scientific background and rationale for the investigation being reported	Pages 5–6
	3	State specific objectives, including any prespecified hypotheses	Pages 5–6
Methods	4	Present key elements of study design early in the paper	Pages 6
	5	Describe the setting, locations, and relevant dates, including periods of recruitment, exposure, follow-up, and data collection	Pages 6–7<br>Figure 1
	6(a)	Give the eligibility criteria, and the sources and methods of selection of participants. Describe methods of follow-up	Pages 6<br>Figure 1
	6(b)	For matched studies, give matching criteria and number of exposed and unexposed	Not applicable
	7	Clearly define all outcomes, exposures, predictors, potential confounders, and effect modifiers. Give diagnostic criteria, if applicable	Pages 7–9
	8	For each variable of interest, give sources of data and details of methods of assessment (measurement). Describe comparability of assessment methods if there is more than one group	Pages 7–9
	9	Describe any efforts to address potential sources of bias	Pages 7–9
	10	Explain how the study size was arrived at	Pages 6<br>Figure 1

Section	Item No.	Recommendation	Page(s)
	11	Explain how quantitative variables were handled in the analyses. If applicable, describe which groupings were chosen and why	Pages 7–9
	12(a)	Describe all statistical methods, including those used to control for confounding	Pages 7–9
	12(b)	Describe any methods used to examine subgroups and interactions	Pages 7–9
	12(c)	Explain how missing data were addressed	Pages 7–9
	12(d)	If applicable, explain how loss to follow-up was addressed	Pages 7–9
	12(e)	Describe any sensitivity analyses	Pages 7–9
Results	13(a)	Report numbers of individuals at each stage—eg numbers potentially eligible, examined for eligibility, confirmed eligible, included in the study, completing follow-up, and analysed	Pages 10–12
	13(b)	Give reasons for non-participation at each stage	Pages 10–12<br>Figure 1
	13(c)	Consider use of a flow diagram	Figure 1
	14(a)	Give characteristics of study participants and information on exposures and potential confounders	Pages 10–12<br>Table 1
	14(b)	Indicate number of participants with missing data for each variable of interest	Table 1
	14(c)	Summarise follow-up time	Page 12–13
	15	Report numbers of outcome events or summary measures over time	Pages 12
	16(a)	Give unadjusted estimates and, if applicable, confounder-adjusted estimates and their precision. Make clear which confounders were adjusted for and why they were included	Pages 12–15
	16(b)	Report category boundaries when continuous variables were categorized	Pages 15

Section	Item No.	Recommendation	Page(s)
	16(c)	If relevant, consider translating estimates of relative risk into absolute risk for a meaningful time period	Not applicable
	17	Report other analyses done—eg analyses of subgroups and interactions, and sensitivity analyses	Pages 12–15
Discussion	18	Summarise key results with reference to study objectives	Pages 15-16
	19	Discuss limitations of the study, taking into account sources of potential bias or imprecision. Discuss both direction and magnitude of any potential bias	Pages 18-19
	20	Give a cautious overall interpretation of results considering objectives, limitations, multiplicity of analyses, results from similar studies, and other relevant evidence	Pages 16–19
	21	Discuss the generalisability (external validity) of the study results	Pages 18-19
Other information	22	Give the source of funding and the role of the funders for the present study and, if applicable, for the original study on which the present article is based	Pages 20

*Page numbers refer to the submitted manuscript file reviewed for this checklist. "Not applicable" is used where the STROBE item does not apply to this cohort study.
